# Genetic divergence between isolated populations of the North Island New Zealand Rifleman (*Acanthisitta chloris granti*) implicates ancient biogeographic impacts rather than recent habitat fragmentation

**DOI:** 10.1002/ece3.7358

**Published:** 2021-05-04

**Authors:** Sarah J. Withers, Stuart Parsons, Mark E. Hauber, Alistair Kendrick, Shane D. Lavery

**Affiliations:** ^1^ School of Biological Sciences Private Bag 92019 Auckland Mail Centre The University of Auckland Auckland New Zealand; ^2^ School of Biology and Environmental Science Queensland University of Technology Brisbane QLD Australia; ^3^ Department of Evolution, Ecology, and Behavior School of Integrative Biology University of Illinois Urbana‐Champaign IL USA; ^4^ Institute of Marine Science Private Bag 92019 Auckland Mail Centre The University of Auckland Auckland New Zealand

**Keywords:** *Acanthisitta chloris granti*, biogeography, Cockayne's Line, genetic divergence, Passerine, phylogeography, Rifleman, Taupo Line

## Abstract

This research investigates the extent and causal mechanisms of genetic population divergence in a poorly flighted passerine, the North Island Rifleman or Titipounamu (*Acanthisitta chloris granti*). While this species has a historically widespread distribution, anthropogenic forest clearance has resulted in a highly fragmented current distribution. We conducted analyses of mitochondrial DNA (COI and Control Region) and 12 nuclear DNA microsatellites to test for population divergence and estimate times of divergence. diyabc and biogeobears were then used to assess likely past dispersal scenarios based on both mtDNA and nDNA. The results reveal several significantly divergent lineages across the North Island of New Zealand and indicate that some populations have been isolated for extensive periods of time (0.7–4.9 mya). Modeling indicated a dynamic history of population connectivity, with a drastic restriction in gene flow between three geographic regions, followed by a more recent re‐establishment of connectivity. Our analyses indicate the dynamic influence of key geological and climatological events on the distribution of genetic diversity in this species, including support for the genetic impact of old biogeographic boundaries such as the Taupo Line and Cockayne's Line, rather than recent anthropogenic habitat fragmentation. These findings present a rare example of an avian species with a genetic history more like that of flightless taxa and so provide new general insights into vicariant processes affecting populations of passerines with limited dispersal.

## Graphical abstract

This research uncovers significant genetic divergence between populations of a basal passerine with restricted dispersal potential, New Zealand's North Island Rifleman. These results demonstrate the impact of ancient biogeographic events on the distribution of this species and present a rare example of an avian species with a genetic history more like that of flightless taxa. This work provides new insights into vicariant processes affecting populations of passerines with limited dispersal more broadly.

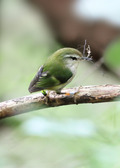

## 1 INTRODUCTION

Intraspecific phylogeographic analyses provide vital information on the distribution of genetic diversity and levels of genetic connectivity across a species’ distribution (Bermingham & Moritz, 1998; Frankham et al., 2002). Molecular indices of diversity may reveal hidden patterns of diversification among populations that are not necessarily evident using phenotypic measures (e.g., Burbridge et al., 2003; Daugherty et al., 1990) and may inform on patterns of historical gene flow. Such studies provide data regarding patterns of individual movement, population‐level gene flow, and potential processes of speciation. The same data also provide essential information to conservation managers. Species that appear widespread may in fact be represented by a series of isolated sub‐populations with varying demographic and diversity characteristics, and their genetic and phenotypic diversity may therefore be at higher risk of extinction than they first appear (Frankham et al., 2002; Koumoundouros et al., 2009). Small isolated populations with low genetic diversity are at greater risk of the effects of inbreeding, genetic drift, and potentially extinction, each of which are critical contributors to species‐level evolutionary and management processes (Bakker et al., 2010; Frankham, 2005; Frankham et al., 1999, 2002). The analysis of population genetic variation is particularly important for less dispersive species. In the presence of geographic barriers to gene flow, species with high dispersal potential may be able to surmount these barriers, whereas sedentary species or species with limited dispersal potential are at greater risk of population isolation, and therefore genetic fragmentation (e.g., Claramunt et al., 2012).

**FIGURE 1:**
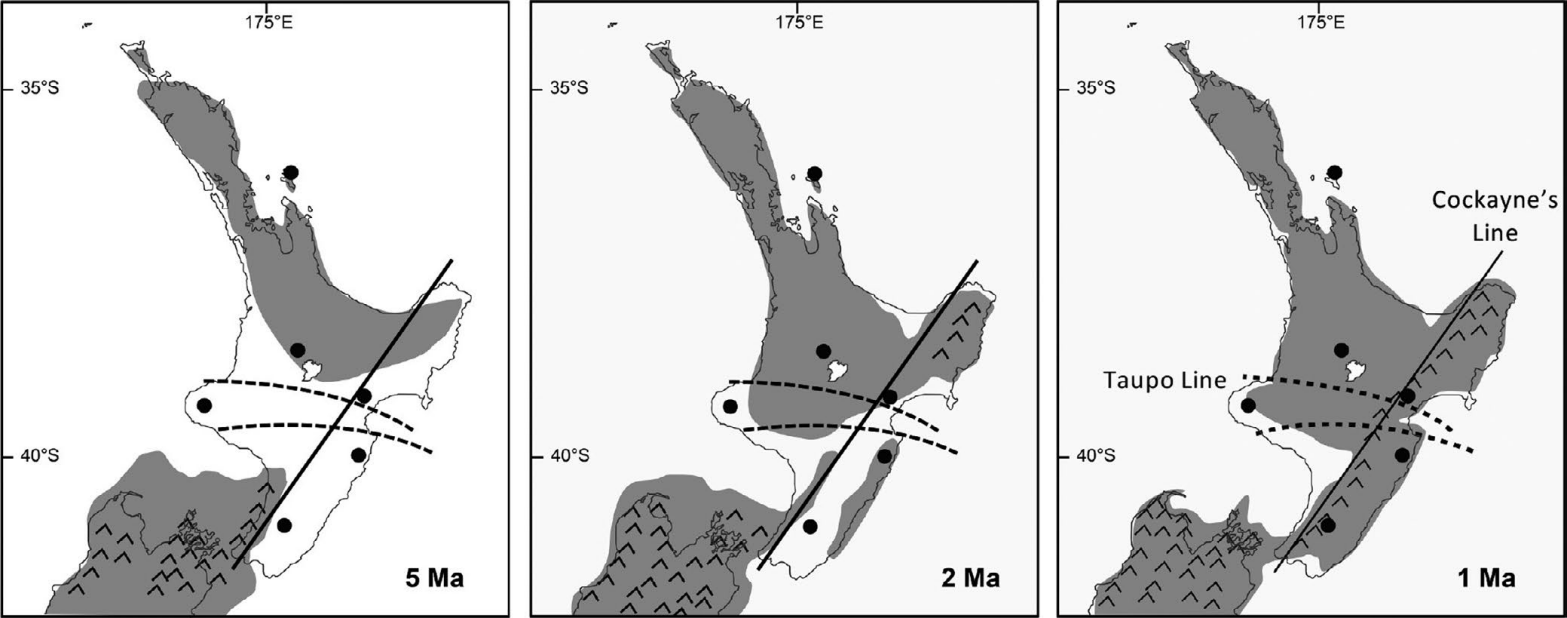
A summary of the significant geological events impacting the distribution of land species in the North Island of New Zealand over three time periods, including the marine submersion of the lower North Island creating the Taupo Line (from McGlone, 1985; Wardle, 1963 (upper), and Rogers & McGlone, 1989 (lower)), and uplift of the axial ranges creating Cockayne's Line (Cockayne, 1911). Sampling locations for this study are also shown

New Zealand's geological and human history makes it a particularly interesting place for phylogeographic studies of terrestrial species, especially those with low dispersal potential (Cooper & Cooper, 1995; Cooper & Millener, 1993; Goldberg et al., 2008; Trewick et al., 2017). This dynamic geological history includes extensive periods of land submersion, montane uplift, and volcanism, as well as repeated glacial cycles, all of which have significantly modified land connectivity and distributions of terrestrial species (Alloway et al., 2007; Fleming, 1962; Goldberg et al., 2008; McGlone, 2005; McGlone et al., 2001). Geological events dated back millions of years have had some of the most significant effects on both flora and fauna in New Zealand. For example, the lower North Island experienced a drastic reduction in land area during a period of extensive land submersion in the Pliocene, significantly restricting distributions of land species up until 2–3 mya (Bunce et al., 2009; Ellis et al., 2015; McGlone, 1985). Following this period of drastic range restriction, uplift along the tectonic plate boundary created an extensive geological barrier to dispersal for nonflighted land species (Cockayne, 1911; Ellis et al., 2015). A summary of the region's most important paleogeographic events is presented in Figure 1 and addressed in more detail in the Discussion. More recently, anthropogenic impacts have included extensive deforestation and the introduction of invasive mammalian predators, which have had significant detrimental impacts on both the abundance and distribution of native species (Gibbs, 2006; Holdaway, 1999; Veitch et al., 2011).

Multiple studies within New Zealand have demonstrated the significant impacts of ancient paleogeographic events on both biogeographic (among‐species) patterns and on phylogeographic (within‐species) patterns of nonavian terrestrial species (Ellis et al., 2015; Trewick et al., 2017). Molecular analyses have generally indicated that phylogeographic patterns are dominated by genetic division between North and South Islands, with relatively little substructure within the North Island itself (summarized in Trewick et al., 2017). Most population divergence within each of the two major islands appears to have occurred relatively recently for birds, due to either anthropogenic factors (e.g., Tracy & Jamieson, 2011) or other geologically recent, postglacial factors (e.g., Dussex et al., 2014; Miller & Lambert, 2006; Weston & Robertson, 2015).

The New Zealand Rifleman (*Acanthisitta chloris*) presents a unique focal species for research into the causal mechanisms of genetic divergence in passerines with lower dispersal potential. The Rifleman is a member of the endemic New Zealand wren family (Acanthisittidae), now considered as the sister group to all other passerines (Barker et al., 2004; Ericson et al., 2002). The family has contained some of the least flighted passerines in the world, including the extinct flightless Lyall's Wren (*Traversia lyalli*; Millener, 1989). The two remaining extant species within the acanthisittid wrens are the Rifleman (*Acanthistta chloris*) and the Rock Wren (*Xenicus gilviventris*). Both species have reduced dispersal ability due to their reduced tail and wing morphology and are therefore likely to have limited long‐range dispersal potential (Higgins et al., 2001). The Rifleman is currently categorized into two insular sub‐species, the North Island Rifleman (*Acanthisitta chloris granti*) and the South Island Rifleman (*Acanthisitta chloris chloris*). The North Island sub‐species was once found throughout the North Island of New Zealand (Gill, 1996; Higgins et al., 2001), but is now limited to a highly fragmented distribution, being restricted predominantly to discontinuous high‐altitude mountain ranges and wooded offshore islands (Robertson et al., 2007). Given the unique phylogenetic position of this species, studies on patterns of past dispersal are particularly informative to both biogeographic and phylogeographic investigations across the world, providing insight into species movements in a dynamic environment.

This investigation aimed to analyze the molecular diversity among geographically separated populations of the North Island sub‐species of Rifleman. We addressed the following questions: (a) Is there evidence for significant genetic divergence among currently fragmented and isolated North Island populations? and (b) Is genetic isolation of populations likely to be related to past geological and climatological factors, or due to more recent anthropogenic forces of change? Given their low dispersal capability, and their previously widespread North Island distribution, we predicted that North Island Rifleman populations would show evidence of genetic population divergence following an isolation‐by‐distance pattern. We used a combination of mtDNA and nDNA to analyze population genetic divergence and to test several different models of past dispersal to determine which model best explains the current genetic distribution for Rifleman.

## 2 MATERIALS AND METHODS

### 2.1 Sample collection

Six locations spread across the North Island of New Zealand were selected for sampling, including Hauturu‐o‐Toi/Little Barrier Island (−36°19′S, 175°07′E), Taranaki National Park (−39°29′S, 174°06′E), Pureora Forest Park (−38°57′S, 175°59′E), the Maungaharuru Ranges (Boundary Stream Mainland Island) (−39°06′S, 176°48′E), Mohi Bush (−39°51′S, 176°54′E), and Tararua Forest Park (−40°86′S, 175°41′E; Figure 1). These areas represented Insular, Western, Central, Eastern Ranges, Eastern Coastal, and Southern locations, respectively. Rifleman were caught within their territories using 24 mm gauge mist‐nets and conspecific lure calls between 2009 and 2012. Blood samples were collected using brachial venipuncture with a 29 gauge sterilized needle and a 20 µl capillary tube. Blood was stored in Queen's lysis buffer (Seutin et al., 1991) and kept at 4°C until DNA extraction. Sample collection was carried out under permits issued by both the New Zealand Department of Conservation (DoC; Banding Permit 2010/025; Regional bird handling permits WE‐25869‐FAU, NO‐26310‐FAU, AK‐27236‐FAU, WK‐28729‐RES, WA‐27986‐FAU) and The University of Auckland Ethics Committee (R762).

### 2.2 DNA extraction

Whole genomic DNA was extracted from blood samples using either standard phenol‐chloroform extraction procedures or using a Qiagen DNEasy Blood and Tissue Kit. The Animal Tissue Modification protocol of the Qiagen Manual was used with the following modifications due to low DNA yields: (a) 160 µl of SET buffer replaced the recommended 200 µl of PBS buffer, (b) 40 µl of Proteinase K replaced the recommended 20 µl, (c) 30 µl of blood in Seutin buffer replaced 10 µl of whole blood, (d) incubation was carried out overnight as opposed to 10 min, and (e) final elution stages were done with two rounds of 100 µl of buffer AE as opposed to 200 µl.

### 2.3 Mitochondrial DNA analyses

Mitochondrial DNA (mtDNA) polymerase chain reaction (PCR) targeted two regions of the mitochondrial genome for amplification. A 751 bp region of the cytochrome oxidase 1 gene (COI) was targeted using the forward primer AWCF1 (Hebert et al., 2004; Patel et al., 2010) (5′‐CGCYTWAACAYTCYGCCATCTTACC‐3′) combined with the reverse primer COIBirdR2 (Hebert et al., 2004; Kerr et al., 2009) (5′‐ACGTGGGAGATAATTCCAAATCCTGG‐3′). COI PCRs were carried out in 25 µl reactions containing 12.83 µl of water, 2.5 µl of 10× reaction buffer, 1.25 mM of MgCl_2_, 2.5 µl of BSA, 0.2 mM of dNTP’s, 0.17 µl of *Taq Ti* polymerase, 1.25 µl of each primer (1.25 µM) and 3 µl of template DNA (10–40 ng/µl). Thermal cycling conditions for amplification involved an initial partial denaturation phase at 94°C for 2 min and 35 cycles of denaturation at 94°C for 30 s, annealing at 57.5°C for 30 s, and extension at 72°C for 30 s, followed by a final extension step at 72°C for 4 min.

Primers targeting an approximately 600 bp region of the 5′ end of the mt Control Region (mtCR/CR) were designed specifically for Rifleman using a published mitochondrial genome from the South Island sub‐species of Rifleman (Accession Number AY325307; Mitchell et al., 2016). The forward primer L16733 (5′‐ACTTGGCACCTCCCCAAGACCA‐3′) was located within the tRNA‐Glu region, and the reverse primer H437 was situated within Domain II of the mtCR (5′‐GGGTTGCTGATTTCTCGTGAG‐3′). PCR reactions contained the same reagent concentrations as for the COI region as detailed above. Thermal cycling conditions were the same as for the COI region, but used an annealing temperature of 57.0°C. PCR products were visualized on a RedSafe‐stained 1.6% agarose gel to check for amplification of single fragments of appropriate length before sequencing. Excess primers and nucleotides were removed from PCR products using the SapEx (Shrimp alkaline phosphatase and exonuclease 1) protocol (Werle et al., 1994), before carrying out cycle sequencing using the Big Dye protocol (Applied Biosystems). Cleanseq (Agencourt) was used to purify products before sequencing on an ABI3130 automated sequencer, using the reverse primer COIBirdR2 for the COI region and the reverse primer H437 for the Control Region.

Sequences were viewed and aligned manually using Geneious Pro (version 5.5.6, Biomatters Inc.). All variable sites were confirmed by visual inspection of chromatograms. Resulting alignments were edited, and low‐quality ends were trimmed to create a 377 bp alignment for the Control Region and a 652 bp alignment for COI. Separate mitochondrial gene alignments were used for subsequent analyses, plus a concatenated dataset was created by combining Control Region and COI sequences. Variable sites were identified using MEGA (version 5.05) to enable manual haplotype designation. Haplotype (h) and nucleotide (π) diversities (Nei, 1987), and measures of departure from neutrality were calculated for individual sample populations using Arlequin v 3.5 (Excoffier & Lischer, 2010). For each separate alignment, the Akaike information criterion (AIC) was used in jModelTest (Guindon & Gascuel, 2003; Posada, 2008) to select the most appropriate nucleotide substitution model for the data (HKY + G). This model was then used to construct phylogenetic trees in Geneious Pro using neighbor‐joining (NJ, 1,000 bootstraps), maximum likelihood (PHYML, 1,000 bootstraps, four substitution rate categories), and Bayesian (MrBayes, 10^7^ chain length, subsample freq. 200, burn‐in 10^6^) methods for visual representation of haplotype relationships.

To visually represent relationships among haplotypes, a haplotype network was created for the COI gene, using a median‐joining algorithm in Network v4.6.1.0 (Flexus Technology Ltd, 2011). Population divergence based on the concatenated COI and Control Region dataset was analyzed in Arlequin using AMOVA, along with pairwise calculations of *F*_ST_ (using haplotype diversities only) and Ф_ST_ (including nucleotide divergences). Statistical adjustment for multiple simultaneous tests was carried out using false discovery rate correction (Benjamini & Yekutieli, 2001). For the COI region, pairwise comparisons between populations were calculated using the Tamura distance method and a gamma level of 0.015, as the closest approximation to the HKY + G model selected by jModel test. Comparisons based on the Control Region divergence data were calculated using the Tamura distance method and a gamma level of 0.306.

beast 2.6.2 was used to estimate divergence dates by a coalescent method, based on the concatenated alignment, using reference sequences (Appendix S1 Table A1; Bouckaert et al., 2014). partitionfinder determined that HKY + G was the most appropriate substitution model for each partition of this dataset, based on a run of 100 million repeats (Guindon & Gascuel, 2003; Lanfear et al., 2012). The alignment was partitioned into four sections (COI positions 1, 2, and 3, and the D‐loop) to allow for differing evolutionary rates. A lognormal relaxed clock, with a gamma site model, was used. Two sets of species divergence dates (Appendix S1 Table A2) were used to calibrate the phylogeny, both based on Mitchell et al. (2016). The maximum calibration times, preferred by Mitchell et al. (2016), are based on a Cretaceous maximum constraint on the divergence of Passeriformes and Psittaciformes, a constraint on the age of the *Acanthisitta* lineage, and not using third codons in their analysis. The minimum calibration times were based on a Paleocene maximum constraint on the root of their passerine tree, no constraint on the age of the *Acanthisitta* lineage, and including third codons in their analysis (Mitchell et al., 2016). MRCA priors were set as normal distributions based on the divergence time means and confidence intervals determined by Mitchell et al. (2016). Each analysis was run with a chain length of 10^8^, and a burn‐in of 10%.

### 2.4 Microsatellite analyses

Twenty microsatellite primers designed for a kinship study in the South Island Rifleman (Preston et al., 2013) were trialed for PCR amplification, and the twelve best performing loci were used in this study. Preston, et al. (2013) identified and characterized thirty‐seven polymorphic microsatellite loci for Kaikoura Tītipounamu. These microsatellites have been used to study kinship in the Kaikoura population (Preston, Briskie, et al., 2013). Primers for twenty of these loci were ordered; after initial trials, twelve loci were selected. These twelve loci amplified more consistently and could be more easily genotyped than the eight rejected loci. Details of the twelve loci are shown in the Appendix S1 (Table A3).

Polymerase chain reaction (PCR) was set up to a total volume of 10 μl, containing 6 μl of water, 1 μl of 10× PCR buffer, 0.5 μl of 25 mM magnesium chloride (MgCl_2_), 1.5 μl of DNA template (10–40 ng/μl), 0.1 μl of the forward primer (10 μM), 0.4 μl of the reverse primer (10 μM), 0.4 μl of fluorescent dye at a concentration of 10 μM (either PET, VIC, 6FAM, or NED; Applied Biosystems), 0.1 μl of dNTP mix (20 mM), and 0.045 μl (0.125 Units) of Platinum^®^ Taq DNA Polymerase (Invitrogen, Life Technologies). PCR cycling was performed using the following amplification temperature profile: initial denaturation at 95°C for 1 min, followed by 40 cycles of 95°C for 15 s, 56°C for 30 s and 72°C for 60 s, and a final extension at 72°C for 5 min. PCR products were electrophoresed on a 1.6% agarose gel, stained with RedSafe, to determine whether amplification was successful. Successfully amplified PCR products were diluted 1:10 in ddH_2_O. 1 μl of diluted PCR product was mixed with 10 μl of HiDi™ formamide (Applied Biosystems) and 0.4 μl GeneScan™ 600 LIZ^®^ Size Standard (Applied Biosystems), in preparation for genotyping. This mix was heat‐shocked at 95°C for 5 min and then cooled at 4°C. Genotyping was performed using an ABI3130xL Genetic Analyzer (Applied Biosystems). Individuals were genotyped based on band length using the microsatellite plugin in Geneious Pro Version 9 (Biomatters Inc.). Genotypes were binned to ensure a constant difference in allele length names of either four (for tetramer repeats) or two (for dimer repeats).

STRUCTURE (version 2.3) was used to estimate the number of divergent population groups in the microsatellite data (Pritchard et al., 2000). A model that allowed for admixture and did not use sampling location as a prior was used. Each run used a 500,000 burn‐in period and 1,000,000 chains for Markov chain Monte Carlo (MCMC). STRUCTURE HARVESTER was used to determine probabilistically what number of populations (*k*) best explains the data (Earl & Vonholdt, 2012). Tests of neutrality and diversity measures were undertaken using micro‐checker (Van Oosterhout et al., 2004), popgenreport (Adamack & Gruber, 2014 ), lositan (Antao et al., 2008 ), and genalex (Peakall & Smouse, 2005 ). Evidence for the effects of a bottleneck on allelic variation within species was tested using the program bottleneck Version 1.2.02 (Cornuet & Luikart, 1996). Population divergence was analyzed using popgenreport and genepop. popgenreport (Adamack & Gruber, 2014) was used to estimate pairwise *F*_ST_, Hedrick's *F′*_ST_ (Hedrick & Goodnight, 2005), and Jost's D (Jost, 2008), the last two of which take into account the downward bias in measuring population divergence caused by high heterozygosity within populations. structure (version 2.3) was used to estimate the number of divergent population groups in the microsatellite data, without location being used as a prior (Pritchard et al., 2000).

### 2.5 Tests of models of past biogeographic history

Initial genetic results indicated that the patterns of mtDNA and nDNA (nuclear DNA) variation observed within the species revealed significant historical divergences among some populations, but that these genetic divergences may have begun to erode in more recent times. As such, we undertook a test of different biogeographic models of past dispersal, in order to determine whether a model that incorporated a recent change in the pattern of dispersal was the best at explaining the patterns of genetic diversity within the species. Three potential dispersal histories were modeled, based on the geographic pattern of genetic variation and the region's known paleogeographic history. The first null model (H0) simulated the scenario where there were no differences in dispersal restrictions among any locations, and there had been no changes over time. The first alternative hypothesis (H1) simulated the scenario suggested by the evidence of three distinct phylogeographic mtDNA lineages—that is, after initial colonization, there were strong dispersal restrictions between the three main regions (Insular, Mainland, and southeastern), and these were maintained until recent times. The second alternative hypothesis (H2) simulated the scenario suggested by the additional evidence of recent sharing of mtDNA and nDNA between Mainland and southeastern populations, but no recent sharing between the two southeastern locations—that is, after initial colonization, there were strong dispersal restrictions between the three main regions, and this was followed by recent breakdown of the barrier between Mainland and southeastern regions, and a new barrier to dispersal between the two southeastern locations (Southern and Eastern Coastal).

These three scenarios were modeled, and tested for fit to the genetic data, using two complementary methods. The first method used diyabc (Cornuet et al., 2010, 2014) to compare the fit of the models to both the mtDNA and nDNA separately and then together, based on summary statistics. Each scenario was modeled through the specification of population divergence or admixture events (detailed in Figure 8). For each set of analyses, 10^7^ datasets were simulated under each scenario, using standard models of microsatellite and mtDNA sequence. The set of summary statistics utilized were Tajima's D, mean sample pairwise differences and *F*_ST_ for mtDNA, and mean genic diversity, *F*_ST_, and (dμ)^2^ distance for microsatellites. The posterior probabilities of each scenario were calculated and compared using the 500 closest simulated datasets in the direct approach, and confidence in the scenario choice was evaluated using 500 datasets from the posterior distribution (Cornuet et al., 2014).

The same three potential models of past dispersal were also compared using biogeobears, which uses a phylogenetic approach to model geographic range distribution (Matzke, 2013). Model weight and parameters are provided in Appendix S1 Table A4. The dated mtDNA phylogeny was used as the basis of comparison, simplified to 14 major haplogroups, and the geographic locations of each haplogroup were recorded. The Rifleman evolutionary period was divided into three periods: 6–3, 3–0.2, and 0.2–0.0 mya, based on hypothesized periods of change in dispersal patterns. The three potential dispersal histories (represented in three time‐stratified dispersal matrices, Appendix S1 Table A5) were compared: H0—no differences in dispersal restrictions among any locations (all dispersal parameters = 1), and no changes over time; H1—after initial colonization (all dispersal parameters = 1), strong dispersal restrictions between the three regions (insular, mainland and southeastern; dispersal = 0.01); H2—after initial colonization (all dispersal parameters = 1), strong dispersal restrictions between the three regions (dispersal = 0.01), followed by recent breakdown of the barrier between mainland and southeastern regions (dispersal = 1), and a new barrier to dispersal between the two southeastern locations (Southern/Tararua Ranges and Eastern Coastal/Mohi Bush, dispersal = 0.01). The three biogeographic models were compared across all possible base dispersal models: DEC, DEC + J, DIVALIKE, DIVALIKE + J, BAYAREALIKE, and BAYAREALIKE + J (Matzke, 2014). The most likely biogeographic model was chosen using AIC or AICc criteria, which both gave identical results across all possible dispersal models. biogeobears is more usually employed in a biogeographic rather than a phylogeographic context, but in our application of this method, the OTUs represent monophyletic lineages that are analogous to the monophyletic populations more usually employed. Here, we were simply modeling the geographic range evolution of these lineages and were not assuming or testing specific biogeographic dispersal models at cladogenetic events.

## 3 RESULTS

### 3.1 Mitochondrial DNA analyses

Mitochondrial DNA sequence data obtained from 90 Rifleman representing six populations across the North Island of New Zealand showed evidence of strong genetic structuring among geographic locations. In the 652 bp COI region, a total of 26 variable nucleotide sites (all involving substitutions) were observed that defined 17 different haplotypes among the six geographic regions (4% mean sequence divergence). The mtCR region contained 46 variable sites that were used to characterize 33 haplotypes in total (12% mean sequence divergence).

Haplotype and nucleotide diversity varied significantly among populations (Table 1). The Insular population contained low haplotype diversity, with all individuals genetically identical for the COI region, but variable for the CR. While the highest haplotype diversity was found in the Western population, all haplotypes found there were closely related, resulting in relatively low nucleotide diversity. The Southern population had relatively high haplotype diversity and the highest nucleotide diversity. Despite having low levels of haplotype diversity, the Eastern Coastal population was characterized by comparatively high nucleotide diversity.

**TABLE 1:** Measures of population genetic diversity for mitochondrial Cytochrome Oxidase I and Control Region sequences, and 12 microsatellite loci from Rifleman populations across the North Island

Pop	mtCOI					mtCR					nDNA		
	*n*	Haplotype Diversity (h ± *SD*)	Nucleotide Diversity (π% ± *SD*)	Tajima's D	Fu's F	*n*	Haplotype Diversity (h ± *SD*)	Nucleotide Diversity (π% ± *SD*)	Tajima's D	Fu's F	*n*	H	Allelic Richness
Insular	21	0.00 ± 0.00	0.00 ± 0.0	0	0	21	0.66 ± 0.08	0.50 ± 0.3	1.57	1.64	24	0.586	1.55
Western	25	0.77 ± 0.07	0.17 ± 0.1	−1.20	**−3.85**	22	0.92 ± 0.03	0.80 ± 0.5	−0.96	**−4.65**	26	0.787	1.82
Central	9	0.50 ± 0.13	0.08 ± 0.1	0.99	0.85	9	0.86 ± 0.09	0.89 ± 0.6	0.02	0.28	10	0.803	1.81
Eastern Ranges	16	0.70 ± 0.05	0.23 ± 0.2	1.91	1.98	15	0.71 ± 0.08	0.69 ± 0.4	−0.19	2.04	33	0.721	1.78
Eastern Coastal	8	0.25 ± 0.18	0.42 ± 0.3	**−1.75**	4.50	8	0.56 ± 0.13	1.13 ± 0.7	**−2.10**	4.59	12	0.808	1.71
Southern	11	0.60 ± 0.15	0.83 ± 0.5	0.86	3.54	11	0.93 ± 0.08	2.68 ± 1.6	0.06	0.94	24	0.811	1.84

Note

Significant Tajima's D (*p* < .05) and Fu's *F* (*p* < .02) are highlighted in bold.

**FIGURE 2:**
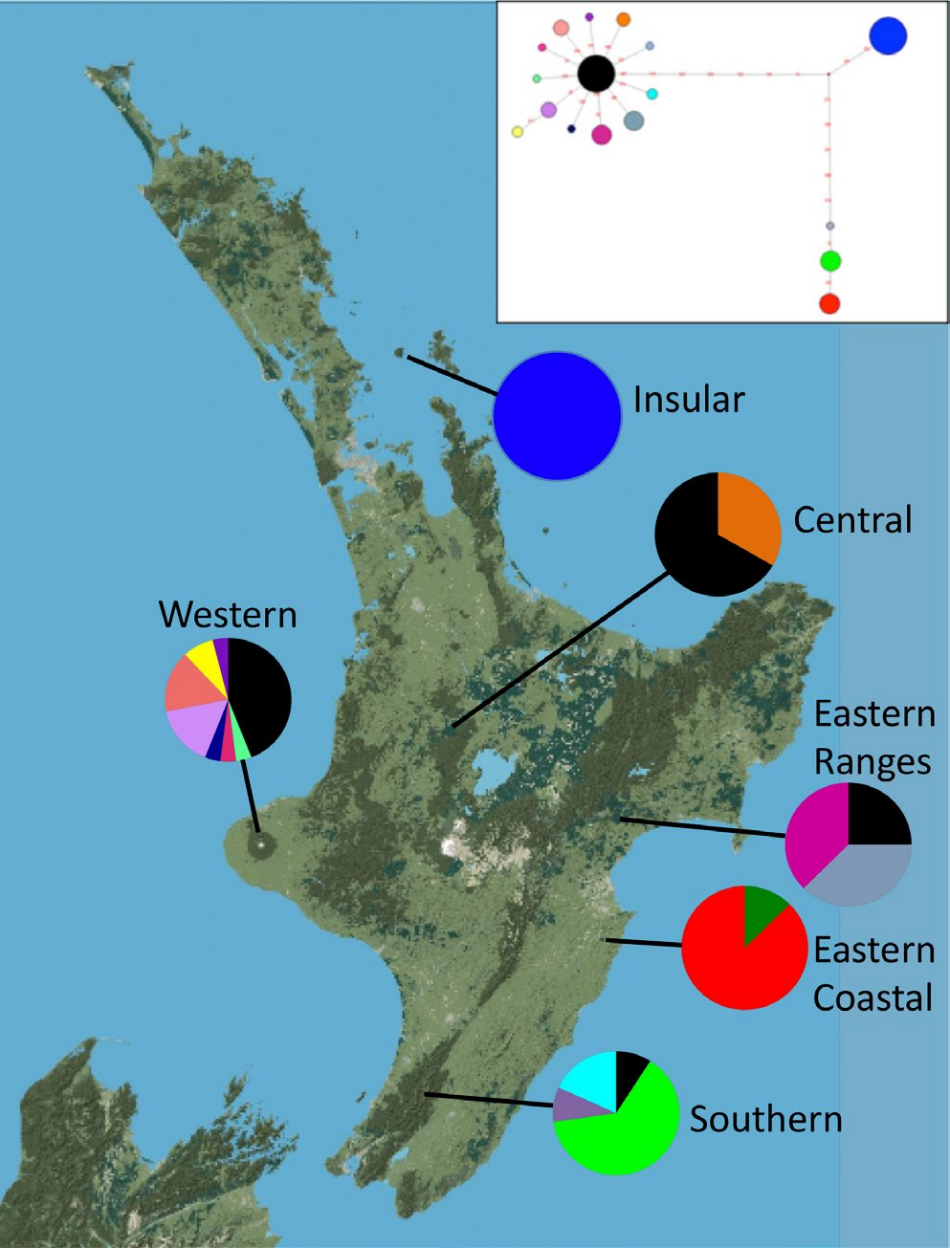
North Island sampling sites of Rifleman and geographic distribution of COI haplotypes. Only a single haplotype is shared across multiple populations. Inset shows the COI haplotype network with identical colors representing haplotypes

Few haplotypes were shared among locations for both the COI and Control Region. The two mitochondrial regions displayed slightly different geographic patterns and were thus analyzed both separately and together in a concatenated dataset. Of the 17 haplotypes represented by COI sequences, only a single haplotype was shared between more than one population (Figure 2). The most common haplotype was found across the three central North Island populations and the Southern population and lies at a central node in the network, most likely representing an ancestral type (Crandall & Templeton, 1993). Most North Island mainland haplotypes diverged from this ancestral type by only a single substitution (Figure 2, inset). A highly differentiated clade of haplotypes was associated with the Eastern Coastal and Southern populations. Although these two populations are dominated by haplotypes that are highly divergent from other mainland haplotypes, these populations also possess minor haplotypes that are closely related to the major ancestral clade. The Insular population was characterized by a single haplotype which was highly divergent, but intermediary, from all other COI haplotypes.

**FIGURE 3:**
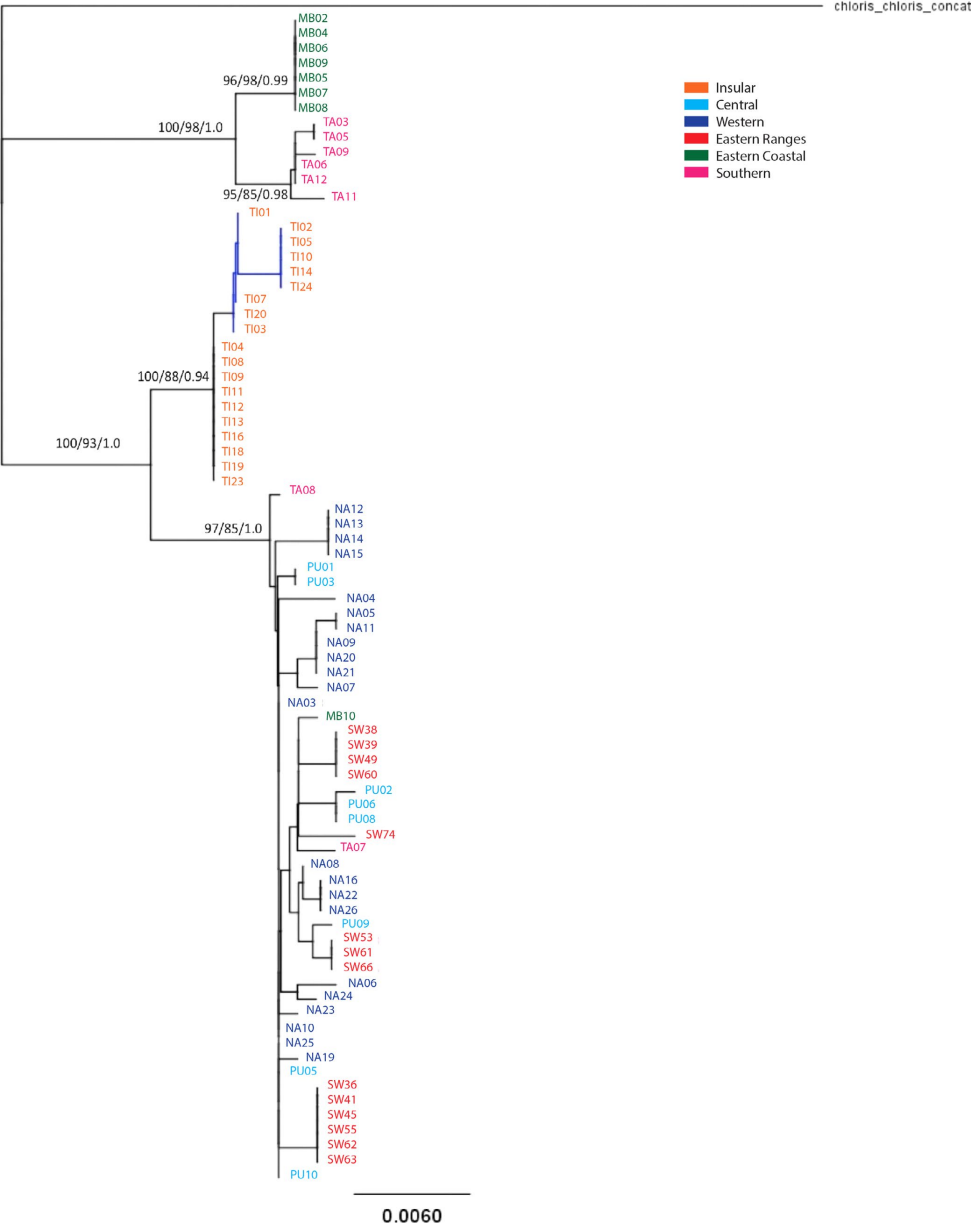
Phylogenetic tree of concatenated COI and Control Region sequences from six populations of North Island Rifleman with South Island Rifleman as an outgroup. Support values are shown for the major clades (neighbor‐joining bootstrap/maximum likelihood bootstrap/Bayesian posterior probability). Concatenated sequences demonstrate three highly divergent lineages across the North Island

The mtCR exhibited high diversity with only one haplotype shared between any two populations (Central and Eastern Range populations). The phylogeographic patterns seen in the mtCR dataset were somewhat different from those seen in COI, but were highly similar to those displayed in the concatenated dataset, and so only the concatenated data are shown here and are best visualized in a phylogenetic tree (Figure 3) due to the large number of haplotypes. Three distinct clades were evident in the phylogenetic tree of the concatenated COI and mtCR dataset, and were supported by all analyses (Figure 3). The three genetically divergent and largely geographically separated clades included one containing all Insular individuals (hereafter referred to as the “Insular clade”), another comprising the majority of the Eastern Coastal and Southern individuals (hereafter referred to as the “southeastern clade”), and the third comprising all remaining sequences from the mainland populations (hereafter referred to as the “Mainland clade”).

The concatenated dataset was used to derive overall population divergence statistics. The overall *F*_ST_ and Ф_ST_ values both showed highly significant isolation among all populations (*F*_ST_ = 0.20, *p* < .0001; Ф_ST_ = 0.74, *p* < .0001). Pairwise population comparisons were all significant at the *p* < .05 level for both *F*_ST_ and Ф_ST_ (Table 2). The Insular population showed the highest levels of divergence for all pairwise comparisons with mainland populations. The Southern and Eastern Coastal populations also had high levels of Ф_ST_ divergence compared to other mainland populations (Table 2). Divergences between the three central mainland sites were lower, but still significant.

**TABLE 2:** Population (*n*) pairwise *F*_ST_ values (below diagonal) and Ф_ST_ values (above diagonal) for the concatenated COI and CR dataset

Population	Insular	Western	Central	Eastern Ranges	Eastern Coastal	Southern
Insular (17)		0.83*	0.90*	0.84*	0.90*	0.80*
Western (20)	0.20*		0.16*	0.24*	0.84*	0.73*
Central (8)	0.24*	0.09*		**0.17**	0.82*	0.65*
Eastern Ranges (15)	0.30*	0.15*	0.18*		0.81*	0.68*
Eastern Coastal (8)	0.33*	0.18*	0.21*	0.28*		**0.24**
Southern (8)	0.23*	0.07*	**0.09**	0.17*	0.20*	

Note

All values were significant (*p* < .05, bold); values significant after FDR correction for multiple tests (*p* < .015) are asterisked.

**FIGURE 4:**
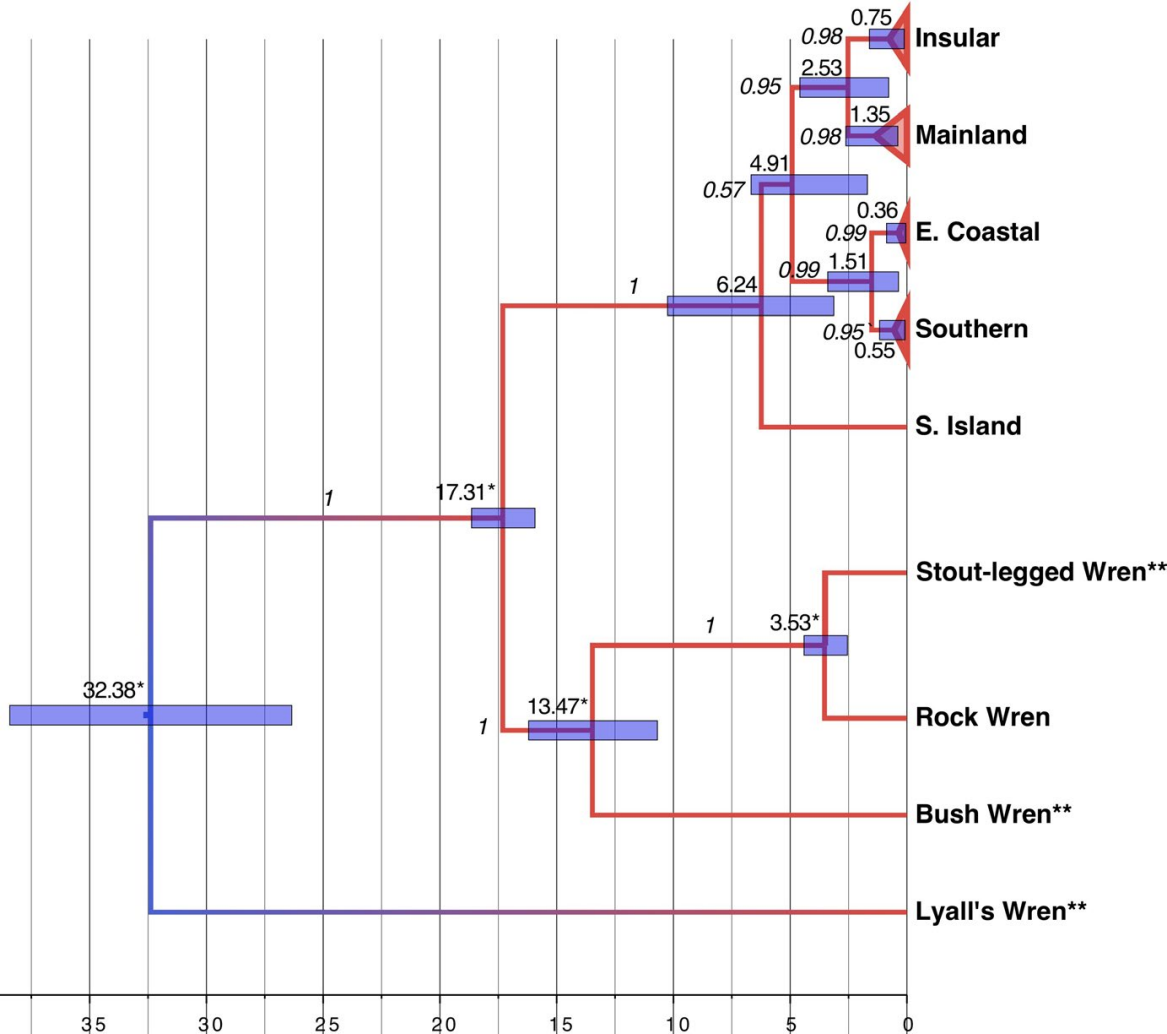
Estimated times of divergence among Rifleman mtDNA lineages and across additional Acanthisittid lineages. Divergence times are estimated using beast analysis and assuming species divergence times based on preferred Maximum Calibration times from Mitchell et al. (2016). Individuals are collapsed within each clade. Divergence times (in mya) are shown above bars representing their 95% HPD (highest posterior density). Posterior probability of node support is shown in italics on branches. Tree lineages are colored by evolutionary rate, ranging from blue (slowest) to red (fastest). *Divergence times at these nodes were set as priors based on Mitchell et al. (2016). **Extinct species

Estimated MRCA (most recent common ancestor) and population divergence times varied depending on the assumptions incorporated in the divergence dating analyses (Table 3). Using the maximum and minimum divergence dates previously calculated from all closely related species (Mitchell et al., 2016), we calculated maximum and minimum node ages for each major clade (Table 3; Figure 4). The Insular clade was estimated to have diverged from the Mainland clade between 1.2 and 2.5 million years ago (mya). Within the southeastern clade, the Eastern Coastal population diverged from the Southern population only 0.7 to 1.5 mya. The earliest North Island divergence, between the south eastern clade and all other populations, was dated at a maximum of 4.9 mya (1.7–6.7 mya). The most recent estimated age of the MRCA from each of the major clades was for the Eastern Coastal clade, between 0.18 and 0.35 mya (Table 3).

**TABLE 3:** Mean node age estimates (mya) and 95% Highest Posterior Densities inferred using BEAST for maximum and minimum sets of parameters

MRCA of clade	Max. estimate		Min. estimate	
	Node ages	95% HPD	Node ages	95% HPD
All Rifleman	6.23	[3.13, 10.25]	2.73	[1.15, 4.55]
North Island	4.91	[1.69, 6.67]	N/A	
Insular + Mainland	2.52	[0.78, 4.59]	1.21	[0.38, 2.16]
Eastern Coastal + Southern	1.50	[0.36, 3.39]	0.72	[0.14, 1.54]
Mainland	1.34	[0.39, 2.62]	0.65	[0.20, 1.25]
Insular	0.75	[0.11, 1.61]	0.39	[0.07, 0.79]
Southern	0.55	[0.08, 1.17]	0.26	[0.03, 0.56]
Eastern Coastal	0.35	[0.05, 0.87]	0.18	[0.02, 0.43]

Note

N/A: this clade did not appear under this set of assumptions.

### 3.2 Microsatellite analyses

No microsatellite loci were found to depart from neutral expectations for tests of selection or departure from Hardy–Weinberg equilibrium. The population with the lowest observed heterozygosity (0.586) and allelic richness (1.55) was the Insular population, which also has low mtDNA diversity. As with the mitochondrial diversity indices, the Southern, Western, and Central mainland populations are the most diverse populations, for both heterozygosity (0.787–0.811) and allelic richness (1.81–1.84). Moreover, despite having very low mtDNA haplotype diversity, the Eastern Coastal population has average nDNA allelic diversity (1.71) and relatively high heterozygosity (0.808). Based on the Bottleneck results, four populations (Insular, Eastern Ranges, Western, and Southern) showed significant evidence for the presence of a bottleneck under at least one model of evolution (Cristescu et al., 2010).

**FIGURE 5:**
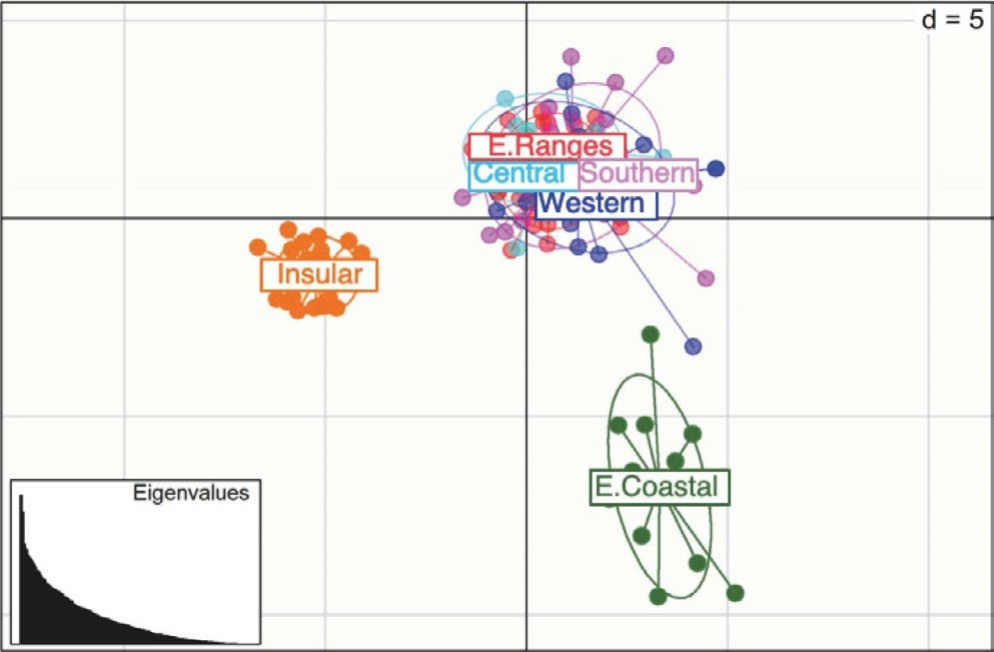
Principal coordinates analysis of microsatellite distances among populations including Insular, Western, Central, Eastern Ranges, Eastern Coastal, and Southern populations

There was a high level of population divergence detected among all populations (*F*_ST_ = 0.181, *p* < .001). Hedrick's *F′*_ST_, which is the *F*_ST_ corrected for within‐population variation, was 0.657 across all loci (Table  4). All pairwise *F*_ST_ and *F′*_ST_ values were still significant after FDR correction for multiple tests (critical value 0.012) (Tables 5 and 6). The largest divergences in pairwise *F′*_ST_ were between the Insular and all other populations. The smallest *F′*_ST_ values were found among the central and southern populations, specifically Central, Eastern Ranges, Western, and Southern. These patterns were reflected in the PCoA analysis (Figure 5), where three groupings clearly emerge: Insular, Eastern Coastal, and the remaining populations.

**TABLE 4:** Microsatellite *F*_ST_ and *F′*_ST_ for each of the twelve loci, and overall

Locus	*F*_ST_	*F′*_ST_
Ach003	0.303	0.783
Ach008	0.073	0.311
Ach010	0.098	0.760
Ach011	0.115	0.668
Ach012	0.210	0.773
Ach014	0.114	0.512
Ach018	0.230	0.899
Ach019	0.140	0.646
Ach024	0.080	0.527
Ach026	0.156	0.710
Ach027	0.093	0.560
Ach028	0.184	0.631
All loci	0.181	0.657

Note

All values are highly significant (*p* < .001).

**TABLE 5:** Pairwise Microsatellite *F*_ST_ values for six populations

	Insular	Central	Eastern ranges	Western	Eastern coastal
Central	0.1799				
Eastern ranges	0.1776	0.0453			
Western	0.1702	0.0302	0.0407		
Eastern Coastal	0.2715	0.1376	0.1337	0.1108	
Southern	0.1818	0.0392	0.0437	0.0246	0.1106

Note

All values were highly significant (*p* < .001). All values were still significant after FDR correction for multiple tests (critical value 0.012).

**TABLE 6:** Pairwise microsatellite *F′*_ST_ values for six populations

	Insular	Central	Eastern ranges	Western	Eastern coastal
Insular (24)					
Central (10)	0.54				
Eastern Ranges (33)	0.54	0.24			
Western (26)	0.55	0.19	0.21		
Eastern Coastal (12)	0.72	0.62	0.56	0.51	
Southern (24)	0.60	0.26	0.24	0.16	0.54

Note

All values were significant (*p* < .05) after FDR correction for multiple tests.

**FIGURE 6:**
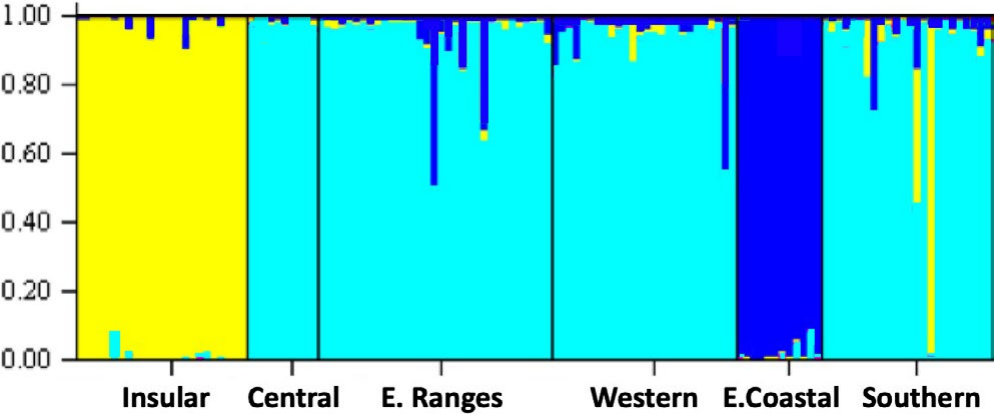
structure results for *k* = 3 groupings

**FIGURE 7:**
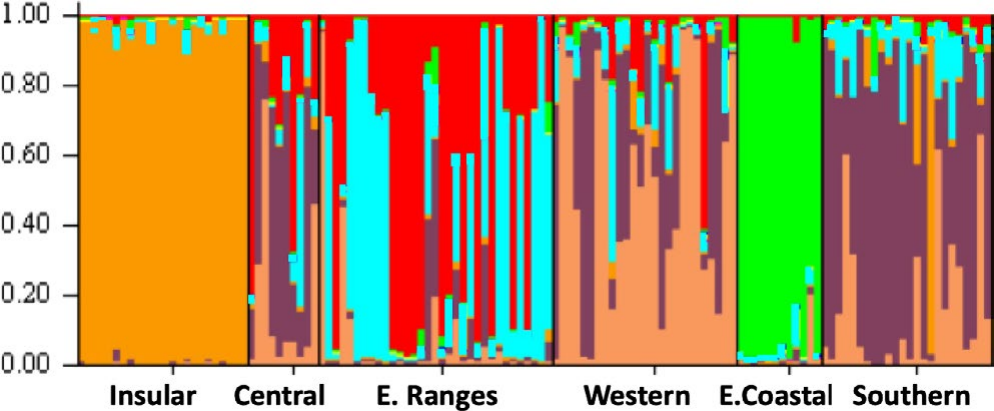
structure results for *k* = 6 groupings

In the Structure analyses, at *k* = 3 the same three clear groupings as above were observed (Figure 6). The putative groupings of individuals correspond very closely to the populations of origin, especially considering that sampling location was not used as a prior. However, these findings differ from the mitochondrial findings, which placed the Southern and Eastern Coastal populations together (the southeastern clade), to the exclusion of the Central, Eastern Ranges, and Western populations (the Mainland clade). Structure Harvester indicated that the number of clusters most supported by the available evidence is *k* = 6. At *k* = 6, the Insular and Eastern Coastal populations stand out as distinct clusters, as for *k* = 3 (Figure 7). All remaining mainland populations appear somewhat distinct, but share genetic clusters among them.

In summary, the microsatellite population divergence indices, PCoA and Structure analyses all indicate that the six populations fall into three significantly different genetic clusters: Insular, Eastern Coastal, and a Central‐Southern North Island grouping (Central, Eastern Ranges, Western, and Southern populations).

### 3.3 Tests of models of past biogeographic history

The combined genetic results above revealed significant historical divergences in both mtDNA and nDNA among the Insular, Mainland, and southeastern regions. However, the mtDNA data also suggested recent sharing of mtDNA between the Mainland and Eastern Coastal populations, and between the Mainland and Southern populations (most evident in COI network, Figures 2 and 3). A similar, recent gene flow was also suggested by the nDNA data, which revealed considerable recent sharing of DNA between the Mainland and Southern populations (most evident in the PCoA, Figure 5). Together, these data suggested that historical genetic divergences among the Mainland and southeastern regions may have begun to erode in more recent times. At the same time, although the Eastern Coastal and Southern populations clearly had a common mtDNA origin, the current genetic divergence between them suggests that a recent barrier to mtDNA gene flow had developed between them (Figures 2 and 3). Overall, the genetic data suggest that the pattern of gene flow among populations has changed in recent times. As such, we undertook a test of different biogeographic models of past dispersal, in order to determine whether a model that incorporated a recent change in the pattern of dispersal was the best at explaining the patterns of genetic diversity within the species.

**FIGURE 8:**
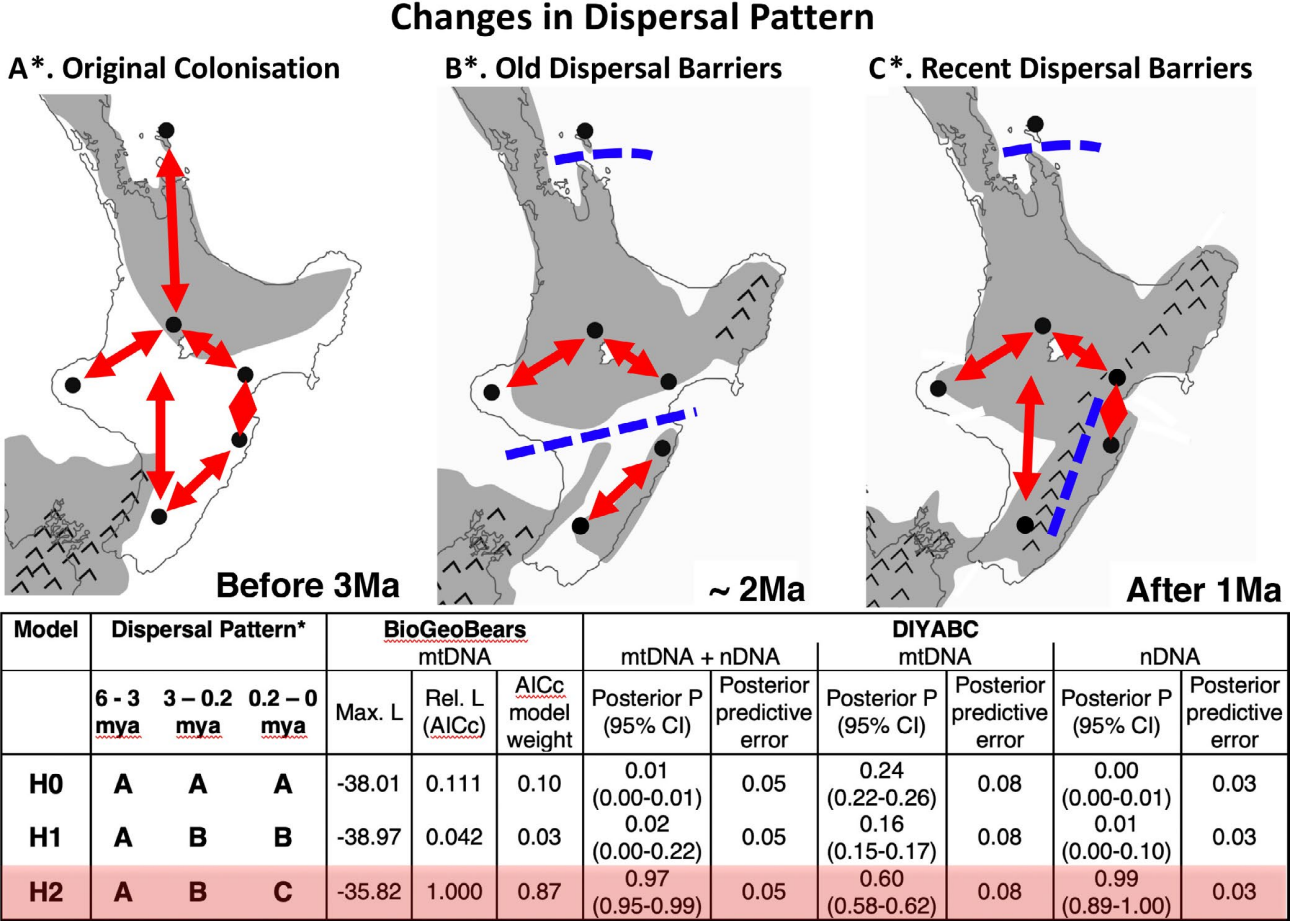
Likelihood of potential changes in dispersal pattern in Rifleman over time modeled using BioGeoBears and DIYABC. The three models of dispersal compared were: H0—null; H1—after colonization, only barriers between Insular, Mainland and southeastern regions; H2—including recent breakdown of barrier between Mainland and southeast. Model H2 was most likely. *A, B, C refer to illustrated patterns. Likelihoods derived from biogeobears analysis of mtDNA, posterior probabilities derived from diyabc analysis of mtDNA and nDNA

The comparison of three potential models of biogeographic dispersal over time (suggested by the genetic data and the region's known paleogeographic history) using the summary statistic approach of diyabc (Figure 8) showed that model H1 (dispersal restrictions only between the Insular, Mainland and southeastern regions) was not more probable than the null model H0 (no differences in dispersal restrictions among any locations, and no changes over time). The model with much higher probability was H2 (after initial colonization, strong dispersal restrictions between the three regions, followed by recent breakdown of the barrier between the Southern population and other mainland populations, and a new barrier between the two southern locations; Figure 8). This was supported by both the mtDNA and nDNA, but most strongly by the nDNA, and there was a low predicted error in these probabilities.

Comparison of the same three models of dispersal over time, using the mtDNA phylogenetic approach of biogeobears, also showed that model H1 did not confer greater likelihood on the data over the null model H0, and that the model that conferred much higher likelihood across all biogeographic models was H2 (Figure 8). Model H2 had the highest likelihood for all dispersal models (using all six basic dispersal assumptions) and together garnered 86.6% of the model weight across all variants (Figure 8).

Together, these results mean that the timing and direction of likely dispersal events correspond far better with the hypothesized history of model H2, than with H0 or H1, although a different model could exist that better fits the data.

## 4 DISCUSSION

This investigation into levels of mitochondrial and nuclear genetic variation among extant Rifleman populations demonstrates the potential impact of ancient biogeographic events on passerines with reduced dispersal potential. We found strong and consistent evidence for major restrictions to gene flow among all sampled Rifleman populations. Dating estimates revealed a complex pattern of dynamic historical connectivity between currently isolated habitat fragments.

Our mitochondrial analyses consistently indicated that Rifleman populations across the North Island of New Zealand contain three deeply divergent mtDNA clades, comprised of the Insular (Little Barrier Island/Hauturu‐o‐Toi population), the southeastern (Tararua Ranges and Mohi Bush populations), and the mainland (remainder of the mainland populations) clades. Although the nDNA data largely concur with this, it does suggest a more recent and closer relationship between the Southern (Tararua Ranges) and central mainland populations (see below). The significant levels of inter‐population divergence from our analyses of both mitochondrial and nuclear DNA indicate that North Island Rifleman populations have been separated for extensive periods of time, consistent with geological impacts stretching back into the Pliocene.

Our estimates of the divergence times among populations are likely to have some error associated with them. Firstly, they are derived from the mtDNA, which is effectively a single locus, and thus subject to considerable stochastic error (Edwards & Beerli, 2000). Secondly, the estimated divergence times are for the mtDNA lineages, which are expected to be earlier than the divergences of the population themselves (Edwards & Beerli, 2000). Thirdly, the Bayesian method of deriving the mtDNA divergence dates from calibration points in the phylogeny may tend to over‐estimate recent dates, particularly when the calibration points are from deeper in time. Thus, our estimates should be considered as maximum divergence times and interpreted in that context. At the same time, the estimates are based on a very confident phylogeny, using a broad range of well‐established calibration points from the most closely related species, and that include the divergence period of interest (Figure 4). As such, we believe our divergence time estimates are the most reliable currently available.

Given those provisos, the genetic separation of the Insular and all mainland sites appears to substantially predate human occupation of the New Zealand archipelago (approx. 1,000 years ago) and is consistent with the long geological isolation of this volcanic island (Hauturu‐o‐Toi/Little Barrier), which is estimated to have emerged approximately 3 mya with subsequent eruptions at approximately 1.6 mya (Lindsay et al., 1999). Land‐bridges to the mainland (approx. 40 km to the south) are likely to have formed at least once during the Pleistocene, when glacial maxima would have caused significant lowering of sea levels (Chapple et al., 2008; Hamilton & Atkinson, 1961; Hare et al., 2008; Lindsay et al., 1999; Turbott, 1961). Nearby mainland populations of Rifleman are now regionally extinct (Robertson et al., 2007); therefore, more fine‐scale patterns of gene flow between Insular and nearby mainland sites could not be investigated. The clear phylogenetic divergence of the Insular population (1.2–2.5 mya) in this analysis suggests that, following colonization from mainland Coromandel, the Insular population was subsequently isolated from the rest of the extant New Zealand populations during the Pleistocene by rising sea levels, and has remained so ever since.

Patterns of population subdivision on the mainland were surprisingly complex. MtDNA data indicated a deep divergence between the southeastern section and the rest of the mainland, dated at approximately 4.9 mya (range 1.7–6.7). Research on a number of species of both plant and animal land taxa has also demonstrated a pattern of population divergence or genetic diversity variation across this divide. Two predominant biogeographic boundaries have been proposed to explain this pattern across the central North Island (Figure 1). The Taupo Line, a line drawn variably between 38.5° and 39.5°S, describes an endemicity and genetic variation North‐South divide for some species of plants (McGlone, 1985; Wardle, 1963) and nonavian land animals (Buckley et al., 2010; Chapple et al., 2008; Marske et al., 2011). Various explanations for the existence of this biogeographic line include sea strait flooding (McGlone, 1985; Rogers & McGlone, 1989; Wardle, 1963), tectonic uplift (McGlone, 1985), volcanism (Chapple et al., 2008; Lloyd, 2003), and Pleistocene glacial cycles (Buckley et al., 2010; Lloyd, 2003). An alternative biogeographic line, Cockayne's Line, describes a barrier effectively splitting the southern North Island into western and eastern regions (Cockayne, 1911; Ellis et al., 2015). Patterns of endemicity, variation, and population divergence aligning with Cockayne's Line have been found in a range of plants and animals and appear to be a direct result of subdivision across the axial ranges, emergent during the Pleistocene (Baker et al., 1995; Buckley et al., 2010; Bunce et al., 2009; Holzapfel et al., 2002; Marshall et al., 2012; Nielson et al., 2011, reviewed in Ellis et al., 2015; Figure 1). Our data on Rifleman are unique in that they appear to show a dynamic pattern of population connectivity among populations of this sub‐species, and we suggest that the distribution of genetic diversity within and among North Island Rifleman populations contains the genetic signal of both the Taupo Line and Cockayne's Line (Figure 8).

One of the predominant explanations for the Taupo Line includes the impact of sea‐level rise creating an extensive sea strait (the Manawatu Strait) during the Pliocene. This strait effectively restricted dry land to a series of islands in the upper region of the North Island and an emergent range in the southernmost part of the current North Island from approximately 23 mya up until as recent as 1.5–2 mya (Bunce et al., 2009; Ellis et al., 2015; McGlone, 1985). Support for the resulting biogeographic effect by the Taupo Line has rarely been found in vertebrates as it may require evidence of divergence prior to 5 mya (Ellis et al., 2015) which may be absent or obscured by more recent genetic patterns (Trewick & Bland, 2011). Rifleman appear to provide an unusual case in that our data demonstrate possible support for the influence of the Manawatu Strait on patterns of genetic connectivity in terrestrial vertebrates in the North Island, with the existence of the divergent southeastern mtDNA clade in the Eastern Coastal (Mohi Bush) and Southern (Tararua Ranges) populations (Figure 8).

As the Manawatu Strait subsided and uplift in the southern North Island occurred, the axial ranges were exposed, connecting the southern North Island and southeastern coastal “islands” approximately 1 mya (Bunce et al., 2009; Ellis et al., 2015; Marske et al., 2009; Trewick & Bland, 2011). This would have allowed Rifleman populations resident in the southern refuge of the North Island (including the Tararua Ranges) to colonize these eastern coastal regions (including Mohi Bush), while areas to the north‐west (including our Mainland populations) may have continued to be isolated due to the emerging ranges, along Cockayne's Line. Our analyses are consistent with this hypothesis, with a much shallower mtDNA divergence time found between the Southern (Tararua Ranges) and Eastern Coastal (Mohi Bush) populations (0.7 to 1.5 mya) than with other mainland populations to the north and west.

In turn, our data also suggest that dispersal patterns have changed substantially through time. Although the three major mtDNA clades indicate very old restrictions to female dispersal between the Insular, Mainland, and southeastern regions, it appears that in more recent times, intermittent dispersal has occurred between central mainland populations and those to the south and southeast. This is evidenced by the small proportion of Mainland clade mtDNA haplotypes that are now found in the Southern (Tararua Ranges) and to a lesser extent in the Eastern Coastal (Mohi Bush) populations and by the microsatellite data that suggest extensive recent dispersal between the Mainland region and the Southern population. Dispersal modeling using both diyabc and biogeobears supports the hypothesis that dispersal patterns have changed in the recent past, with increased gene flow from Mainland to both Southern (Tararua Ranges) and Eastern Coastal (Mohi Bush) populations, accompanied by reduced dispersal between the Southern and Eastern Coastal populations. The nDNA data also suggest that nuclear gene flow has now eroded much of the divergence of the Southern (Tararua Ranges) population, but little of the Eastern Coastal (Mohi Bush) divergence. One reason for this difference between mtDNA and nDNA divergence is likely to be the greater dispersal of males, which will more rapidly break down nDNA divergence among populations. This evidence for changing patterns of dispersal over time concurs with known paleogeographic patterns and may also demonstrate the dynamic nature of forest distribution throughout the late Pleistocene in response to glacial cycles and geological impacts (Buckley et al., 2009; McGlone, 1985; Trewick & Bland, 2011).

Populations in the central and north‐west of the mainland (the Mainland clade) were much more closely related to each other than to the southeastern populations. The shallow phylogenetic differentiation of sequences and the star‐shaped COI haplotype network evident within the Mainland clade are both evidence of more recent population expansion (Dixon, 2011; Rogers & Harpending, 1992), possibly from Pleistocene glacial refugia, and dated here at between 0.6 and 1.3 mya. Nuclear microsatellite data support this close relationship. Glacial cycles during the Pleistocene are likely to have caused mature forest to be restricted to the very north of the North Island during the height of glacial periods, with small, isolated patches of forest refugia elsewhere (Buckley et al., 2009; McGlone et al., 2001), while volcanic episodes caused substantial range disruption through the Central Plateau (Trewick & Bland, 2011). As a forest species, Rifleman are reliant on mature native trees for nesting and foraging (Higgins et al., 2001). Rifleman would have been restricted to these forest refugia during Pleistocene glacial and volcanic periods. Despite the Western, Central, and Eastern Ranges populations all possessing haplotypes from the one mitochondrial clade, contemporary patterns of genetic differentiation appear to indicate a current lack of connectivity between all sampled populations. The three populations shared only a single mtDNA haplotype and showed consistently significant divergence estimates at both mtDNA and nDNA, indicating highly restricted recent connectivity between populations.

The results of this study differ markedly from previous studies on other New Zealand bird species, where fragmented populations within each main island appear to be more recently isolated, due to either recent anthropogenic factors (e.g., Baillie et al., 2014; Tracy & Jamieson, 2011), or postglacial factors (Baillie et al., 2014; Dussex et al., 2014; Miller & Lambert, 2006; Weston & Robertson, 2015). The deep genetic divisions shown in this study are even remarkable when compared to extinct, flightless New Zealand species such as North Island moa (Dinornithiformes) populations, which diverged only recently during Pleistocene glacial cycles (Baker et al., 1995). Worldwide, species divergence patterns in similarly less dispersive groups provide us with insights into how population divergence such as that seen in the rifleman could lead to speciation. In lowland neotropical antpittas (Grallariidae), ongoing species divergence extending back into the Oligocene is likely to have resulted simply from slow and constant diversification over long time scales, with low dispersal acting to maintain boundaries between variants (Carneiro et al., 2018). Similarly, in less dispersive Old World Eurylaimides, ancient vicariance events combined with more recent climatic and geological processes have resulted in species divergence, with historic patterns of gene flow evident due to the lack of dispersal (Moyle et al., 2006). Our data on the Rifleman appear to demonstrate a similar effect in that, due to restrictions in dispersal ability, a lack of extensive gene flow has allowed us to view the long‐term patterns of ancient diversification within this species. Indeed, in a New Zealand context, Rifleman appear to exhibit biogeographic patterns more synonymous with several studies on nonavian taxa as opposed to avian species. Support for the Taupo Line has been found in beetles (Marske et al., 2011), while some native skink species contain deep population divergence hypothesized to result from Pliocene volcanic activity (Chapple et al., 2008). In turn, support for Cockayne's Line, and therefore the impact of Pleistocene mountain uplift, has been found in stick insects (*Clitarchus* sp.; Buckley et al., 2010), geckos (Diplodactylidae; Nielson et al., 2011), cicadas (*Kikihia* sp.; Ellis et al., 2015), and in the plant genus *Dactylanthus* (Holzapfel et al., 2002). Rifleman therefore appear to be a remarkable case among birds in that they show the genetic signal of past dispersal barriers on a scale usually seen only in invertebrates and plants. This study provides a unique perspective on the impact of ancient vicariant processes on the genetic distribution of avian species with reduced dispersal. These results demonstrate how the Rifleman, a species with high antiquity, no near relatives, and low dispersal ability, is an exceptional candidate to provide a glimpse into ancient historical vicariant processes affecting land birds.

## CONFLICT OF INTEREST

There are no conflicts of interest for any author listed in this manuscript.

## AUTHOR CONTRIBUTIONS

**Sarah Withers:** Conceptualization (lead); Data curation (lead); Formal analysis (lead); Funding acquisition (equal); Investigation (lead); Methodology (lead); Validation (lead); Visualization (lead); Writing‐original draft (lead); Writing‐review & editing (equal). **Stuart Parsons:** Conceptualization (equal); Data curation (supporting); Formal analysis (supporting); Funding acquisition (equal); Investigation (supporting); Methodology (supporting); Project administration (equal); Resources (equal); Supervision (lead); Writing‐review & editing (equal). **Mark Hauber:** Conceptualization (equal); Data curation (supporting); Formal analysis (supporting); Funding acquisition (equal); Investigation (supporting); Methodology (supporting); Project administration (equal); Resources (equal); Supervision (lead); Writing‐review & editing (equal). **Alistair Kendrick:** Data curation (equal); Formal analysis (lead); Investigation (equal); Methodology (lead); Visualization (lead); Writing‐original draft (supporting); Writing‐review & editing (supporting). **Shane Lavery:** Conceptualization (equal); Data curation (equal); Formal analysis (lead); Funding acquisition (equal); Investigation (equal); Methodology (equal); Project administration (equal); Resources (equal); Supervision (lead); Validation (equal); Visualization (equal); Writing‐review & editing (lead).

## Supporting information

Appendix S1

## Data Availability

mtDNA and nDNA data are located in GenBank with accession numbers (MW626949‐MW627125).

## References

[ece37358-bib-0001] Adamack, A. T. , & Gruber, B. (2014). PopGenReport: Simplifying basic population genetic analyses in R. Methods in Ecology and Evolution, 5(4), 384–387.

[ece37358-bib-0002] Alloway, B. V. , Lowe, D. J. , Barrell, D. J. A. , Newnham, R. M. , Almond, P. C. , Augustinus, P. C. , Bertler, N. A. N. , Carter, L. , Litchfield, N. J. , McGlone, M. S. , Shulmeister, J. , Vandergoes, M. J. , Williams, P. W. , & NZ‐INTIMATE members . (2007). Towards a climate event stratigraphy for New Zealand over the past 30,000 years (NZ‐INTIMATE project). Journal of Quaternary Science, 22, 9–35.

[ece37358-bib-0003] Antao, T. , Lopes, A. , Lopes, R. J. , Beja‐Pereira, A. , & Luikart, G. (2008). A workbench to detect molecular adaptation based on *F* _ST_‐outlier method. BMC Bioinformatics, 9(323). 10.1186/1471-2105-9-323 PMC2515854 18662398

[ece37358-bib-0004] Baillie, S. M. , Ritchie, P. H. , & Brunton, D. H. (2014). Population genetic connectivity of an endemic New Zealand passerine after large‐scale local extirpations: A model of recolonization potential. Ibis, 156(4), 826–839. 10.1111/ibi.12182

[ece37358-bib-0005] Baker, A. J. , Daugherty, C. H. , Colbourne, R. M. , & McLennan, J. L. (1995). Flightless brown kiwis of New Zealand possess extremely subdivided population structure and cryptic species like small mammals. Proceedings of the National Academy of Science USA, 92, 8254–8258. 10.1073/pnas.92.18.8254 PMC41135 7667277

[ece37358-bib-0006] Bakker, J. , van Rijswijk, M. E. C. , Weissing, F. J. , & Bijlsma, R. (2010). Consequences of fragmentation for the ability to adapt to novel environments in experimental *Drosophila* metapopulations. Conservation Genetics, 11, 435–448. 10.1007/s10592-010-0052-5

[ece37358-bib-0007] Barker, F. K. , Cibois, A. , Schikler, P. , Feinstein, J. , & Cracraft, J. (2004). Phylogeny and diversification of the largest avian radiation. Proceedings of the National Academy of Science, 101, 11040–11045. 10.1073/pnas.0401892101 PMC503738 15263073

[ece37358-bib-0008] Benjamini, Y. , & Yekutieli, D. (2001). The control of false discovery rate under dependency. The Annals of Statistics, 29, 1165–1188.

[ece37358-bib-0009] Bermingham, E. , & Moritz, C. (1998). Comparative phylogeography: Concepts and applications. Molecular Ecology, 7, 367–369. 10.1046/j.1365-294x.1998.00424.x

[ece37358-bib-0010] Bouckaert, R. , Heled, J. , Kuhnert, D. , Vaughan, T. , Wu, C. , Xie, D. , Suchard, M. , Rambaut, A. , & Drummond, A. (2014). BEAST 2: A software platform for Bayesian evolutionary analyses. PLoS Computational Biology, 10, e1003537. 24722319 10.1371/journal.pcbi.1003537 PMC3985171

[ece37358-bib-0011] Buckley, T. R. , Marske, K. A. , & Attanayake, D. (2009). Identifying glacial refugia in a geographic parthenogen using palaeoclimate modelling and phylogeography: The New Zealand stick insect *Argosarchus horridus (*White). Molecular Ecology, 18, 4650–4663. 19840262 10.1111/j.1365-294X.2009.04396.x

[ece37358-bib-0012] Buckley, T. R. , Marske, K. A. , & Attanayake, D. (2010). Phylogeography and ecological niche modelling of the New Zealand stick insect *Clitarchus hookeri* (White) support survival in multiple coastal refugia. Journal of Biogeography, 37, 682–695.

[ece37358-bib-0013] Bunce, M. , Worthy, T. H. , Phillips, M. J. , Holdaway, R. N. , Willerslev, E. , Haile, J. , Shapiro, B. , Scofield, R. P. , Drummond, A. , Kamp, P. J. J. , & Cooper, A. (2009). The evolutionary history of the extinct ratite moa and New Zealand Neogene paleogeography. Proceedings of the National Academy of Sciences, 106, 20646–20651. 10.1073/pnas.0906660106 PMC2791642 19923428

[ece37358-bib-0014] Burbridge, M. L. , Colbourne, R. M. , Robertson, H. A. , & Baker, A. J. (2003). Molecular and other biological evidence supports the recognition of at least three species of brown kiwi. Conservation Genetics, 4, 167–177.

[ece37358-bib-0015] Carneiro, L. , Bravo, G. A. , Aristizabal, N. , Cuervo, A. M. , & Aleixo, A. (2018). Molecular systematics and biogeography of lowland antpittas (Aves, Grallariidae): The role of vicariance and dispersal in the diversification of a widespread Neotropical lineage. Molecular Phylogenetics and Evolution, 120, 375–389. 10.1016/j.ympev.2017.11.019 29233706

[ece37358-bib-0016] Chapple, D. G. , Daugherty, C. H. , & Ritchie, P. A. (2008). Comparative phylogeography reveals pre‐decline population stucture of New Zealand *Cyclodina* (Reptilia: Scincidae) species. Biological Journal of the Linnaean Society, 95, 388–408.

[ece37358-bib-0017] Claramunt, S. , Derryberry, E. P. , Remsen, J. V. Jr. , & Brumfield, R. T. (2012). High dispersal ability inhibits speciation in a continental radiation of passerine birds. Proceedings of the Royal Society of London, Series B, 279, 1567–1574. 10.1098/rspb.2011.1922 22090382 PMC3282344

[ece37358-bib-0018] Cockayne, L. (1911). Observations concerning evolution, derived from Ecological Studies in New Zealand. Transactions of the New Zealand Institute. Plates I‐VIII.

[ece37358-bib-0019] Cooper, A. , & Cooper, R. A. (1995). The Oligocene bottleneck and New Zealand biota: Genetic record of a past environmental crisis. Proceedings of the Royal Society of London. Series B, 261, 293–302. 8587872 10.1098/rspb.1995.0150

[ece37358-bib-0020] Cooper, R. A. , & Millener, P. R. (1993). The New Zealand biota: Historical background and new research. TRENDS in Ecology and Evolution, 8, 429–433. 10.1016/0169-5347(93)90004-9 21236222

[ece37358-bib-0021] Cornuet, J. M. , & Luikart, G. (1996). Description and power analysis of two tests for detecting recent population bottlenecks from allele frequency data. Genetics, 144(4), 2001–2014. 10.1093/genetics/144.4.2001 8978083 PMC1207747

[ece37358-bib-0022] Cornuet, J.‐M. , Pudlo, P. , Veyssier, J. , Dehne‐Garcia, A. , Gautier, M. , Leblois, R. , Marin, J.‐M. , & Estoup, A. (2014). DIYABC v2.0: A software to make approximate Bayesian computation inferences about population history using single nucleotide polymorphism, DNA Sequence and Microsatellite Data. Bioinformatics, 30, 1187–1189. 24389659 10.1093/bioinformatics/btt763

[ece37358-bib-0023] Cornuet, J. M. , Ravigne, V. , & Estoup, A. (2010). Inference on population history and model checking using DNA sequence and microsatellite data with the software DIYABC (v1.0). BMC Bioinformatics, 11, 401. 20667077 10.1186/1471-2105-11-401 PMC2919520

[ece37358-bib-0087] Crandall, K.A. , & Templeton, A.R. (1993). Empirical tests of some predictions from coalescent theory with applications to intraspecific phylogeny reconstruction. Genetics, 134, 959–969. 8349118 10.1093/genetics/134.3.959 PMC1205530

[ece37358-bib-0088] Cristescu, R. , Sherwin, W.B. , Handasyde, K. , Cahill, V. , & Cooper, D.W. (2010). Detecting bottlenecks using BOTTLENECK 1.2.02 in wild populations: the importance of microsatellite structure. Conservation Genetics, 1043–1049.

[ece37358-bib-0024] Daugherty, C. H. , Patterson, G. B. , Thorn, C. J. , & French, D. C. (1990). Differentiation of the members of the New Zealand *Leiolopisma nigriplantare* species complex (Lacertilia: Scincidae). Herpetological Monographs, 4, 61–76. 10.2307/1466968

[ece37358-bib-0025] Dixon, M. D. (2011). Post‐Pleistocene range expansion of the recently imperiled eastern little brown bat (*Myotis lucifugus lucifugus*) from a single southern refugium. Ecology and Evolution, 1(2), 191–200. 22393495 10.1002/ece3.20 PMC3287298

[ece37358-bib-0026] Dussex, N. , Wegmann, D. , & Robertson, B. C. (2014). Postglacial expansion and not human influence best explains the population structure in the endangered kea (*Nestor notabilis*). Molecular Ecology, 23, 2193–2209. 24684223 10.1111/mec.12729

[ece37358-bib-0027] Earl, D. , & vonHoldt, B. (2012). STRUCTURE HARVESTER: A website and program for visualizing STRUCTURE output and implementing the Evanno method. Conservation Genetics Resources, 4, 359–361. 10.1007/s12686-011-9548-7

[ece37358-bib-0028] Edwards, S. V. , & Beerli, P. (2000). Gene divergence, population divergence, and the variance in coalescence time in phylogeographic studies. Evolution, 54, 1839–1854. 11209764 10.1111/j.0014-3820.2000.tb01231.x

[ece37358-bib-0029] Ellis, E. A. , Marshall, D. C. , Hill, K. B. R. , Owen, C. L. , Kamp, P. J. J. , & Simon, C. (2015). Phylogeography of six codistributed New Zealand cicadas and their relationship to multiple biogeographic boundaries suggest a re‐evaluation of the Taupo Line. Journal of Biogeography, 42, 1761–1775.

[ece37358-bib-0030] Ericson, P. G. P. , Christidis, L. , Cooper, A. , Irestedt, M. , Jackson, J. , Johansson, U. A. , & Norman, J. A. (2002). A Gondwanan origin of passerine birds supported by DNA sequences of the endemic New Zealand wrens. Proceedings of the Royal Society of London. Series B, 269, 235–241. 11839192 10.1098/rspb.2001.1877 PMC1690883

[ece37358-bib-0031] Excoffier, L. , & Lischer, H. (2010). Arlequin suite ver 3.5: A new series of programs to perform population genetics analyses under Linux and Windows. Molecular Ecology Resources, 10, 564–567. 10.1111/j.1755-0998.2010.02847.x 21565059

[ece37358-bib-0032] Fleming, C. A. (1962). New Zealand biogeography—A paleontologist's approach. Tuatara, 10(2), 53–107.

[ece37358-bib-0033] Frankham, R. (2005). Genetics and extinction. Conservation Genetics, 7, 879–893.

[ece37358-bib-0034] Frankham, R. , Ballou, J. D. , & Briscoe, D. A. (2002). Introduction to conservation genetics. Cambridge University Press.

[ece37358-bib-0035] Frankham, R. , Lees, K. , Montgomery, M. E. , England, P. R. , Lowe, E. H. , & Briscoe, D. A. (1999). Do population size bottlenecks reduce evolutionary potential? Animal Conservation, 2, 255–260. 10.1111/j.1469-1795.1999.tb00071.x

[ece37358-bib-0036] Gibbs, G. (2006). Ghosts of Gondwana: The history of life in New Zealand. Craig Potton Publishing.

[ece37358-bib-0037] Gill, B. J. (1996). A fossil bone of the Rifleman (*Acanthisitta chloris*) from Cape Reinga. Notornis, 43, 113–114.

[ece37358-bib-0038] Goldberg, J. , Trewick, S. A. , & Paterson, A. M. (2008). Evolution of New Zealand's terrestrial fauna: A review of molecular evidence. Philosophical Transactions of the Royal Society Series B, 363, 3319–3334. 10.1098/rstb.2008.0114 PMC2607375 18782728

[ece37358-bib-0039] Guindon, S. , & Gascuel, O. (2003). A simple, fast, and accurate algorithm to estimate large phylogenies by maximum likelihood. Systematic Biology, 52, 696–704. 10.1080/10635150390235520 14530136

[ece37358-bib-0040] Hamilton, W. M. , & Atkinson, I. A. (1961). Vegetation. In W. M. Hamilton (Ed.), Little Barrier Island (Hauturu), Vol. 137. New Zealand DSIR Bulletin.

[ece37358-bib-0041] Hare, K. M. , Daugherty, C. H. , & Chapple, D. G. (2008). Comparative phylogeography of three skink species (*Oligosoma moco, O. smithi, O. suteri;* Reptilia: Scincidae) in Northeastern New Zealand. Molecular Phylogenetics and Evolution, 46, 303–315. 17911035 10.1016/j.ympev.2007.08.012

[ece37358-bib-0042] Hebert, P. D. N. , Stoeckle, M. Y. , Zemlak, R. S. , & Francis, C. M. (2004). Identification of birds through DNA barcodes. PLoS Biology, 2, e312. 10.1371/journal.pbio.0020312 15455034 PMC518999

[ece37358-bib-0043] Hedrick, P. W. , & Goodnight, C. (2005). A standardized genetic differentiation measure. Evolution, 59(8), 1633–1638. 10.1111/j.0014-3820.2005.tb01814.x 16329237

[ece37358-bib-0044] P. J. Higgins , P. Jin , & K. W. Steele (Eds.). (2001). Handbook of Australian, New Zealand and Antarctic Birds (Vol. 5: Tyrant flycatchers to Chats). Oxford University Press.

[ece37358-bib-0045] Holdaway, R. N. (1999). Introduced predators and avifaunal extinction in New Zealand. In R. D. E. MacPhee (Ed.), Extinctions in near time: Causes, contexts and consequences. Kluwer Academic and Plenum Press.

[ece37358-bib-0046] Holzapfel, S. , Faville, M. Z. , & Gemmill, C. E. C. (2002). Genetic variation of the endangered holoparasite *Dactylanthus taylorii* (Balanophoraceae) in New Zealand. Journal of Biogeography, 29, 663–676. 10.1046/j.1365-2699.2002.00715.x

[ece37358-bib-0048] Jost, L. (2008). GST and its relatives do not measure differentiation. Molecular Ecology, 17(18), 4015–4026. 19238703 10.1111/j.1365-294x.2008.03887.x

[ece37358-bib-0049] Kerr, K. C. R. , Lijtmaer, D. A. , Barreira, A. S. , Hebert, P. D. N. , & Tubaro, P. L. (2009). Probing evolutionary patterns in neotropical birds through DNA barcodes. PLoS One, 4, e4379. 10.1371/journal.pone.0004379 19194495 PMC2632745

[ece37358-bib-0050] Koumoundouros, T. , Sumner, J. , Clemann, N. , & Stuart‐Fox, D. (2009). Current genetic isolation and fragmentation contrasts with historical connectivity in an alpine lizard (*Cyclodomorphus praealtus*) threatened by climate change. Biological Conservation, 142, 992–1002. 10.1016/j.biocon.2008.12.026

[ece37358-bib-0051] Lanfear, R. , Calcott, B. , Ho, S. Y. , & Guindon, S. (2012). PartitionFinder: Combined selection of partitioning schemes and substitution models for phylogenetic analyses. Molecular Biology and Evolution, 29, 1695–1701. 10.1093/molbev/mss020 22319168

[ece37358-bib-0052] Lindsay, J. M. , Worthington, T. J. , Smith, I. E. M. , & Black, P. M. (1999). Geology, petrology, and petrogenesis of Little Barrier Island, Hauraki Gulf, New Zealand. New Zealand Journal of Geology and Geophysics, 42, 155–168. 10.1080/00288306.1999.9514837

[ece37358-bib-0053] Lloyd, B. D. (2003). Intraspecific phylogeny of the New Zealand short‐tailed bat Mystacina tuberculata inferred from multiple mitochondrial gene sequences. Systematic Biology, 52(4), 460–476. 10.1080/10635150390218187 12857638

[ece37358-bib-0054] Marshall, D. C. , Hill, K. B. R. , Marske, K. A. , Chambers, C. , Buckley, T. R. , & Simon, C. (2012). Limited, episodic diversification and contrasting phylogeography in a New Zealand cicada radiation. BMC Evolutionary Biology, 12, 177. 10.1186/1471-2148-12-177 22967046 PMC3537654

[ece37358-bib-0055] Marske, K. A. , Leschen, R. A. B. , Barker, G. M. , & Buckley, T. R. (2009). Phylogeographic and ecological niche modelling implicate coastal refugia and trans‐alpine dispersal of a New Zealand fungus beetle. Molecular Ecology, 18, 5126–5142. 19900173 10.1111/j.1365-294X.2009.04418.x

[ece37358-bib-0086] Marske, K.A. , Leschen, R.A.B. , & Buckley, T.R. (2011). Reconciling phylogeography and ecological niche models for New Zealand beetles: Looking beyond glacial refugia. Molecular Phylogenetics and Evolution(59), 89–102. 10.1016/j.ympev.2011.01.005 21262367

[ece37358-bib-0056] Matzke, N. J. (2013). Probabilistic historical biogeography: New models for founder‐event speciation, imperfect detection, and fossils allows improved accuracy and model testing. Frontiers of Biogeography, 5(4), 242–248.

[ece37358-bib-0057] Matzke, N. J. (2014). Model selection in historical biogeography reveals that founder‐event speciation is a crucial process in Island Clades. Systematic Biology, 63, 951–970. 10.1093/sysbio/syu056 25123369

[ece37358-bib-0058] McGlone, M. S. (1985). Plant biogeography and the late Cenozoic history of New Zealand. New Zealand Journal of Botany, 23, 723–749. 10.1080/0028825X.1985.10434240

[ece37358-bib-0059] McGlone, M. S. (2005). Goodbye Gondwana. Journal of Biogeography, 32, 739–740. 10.1111/j.1365-2699.2005.01278.x

[ece37358-bib-0060] McGlone, M. S. , Duncan, R. P. , & Heenan, P. B. (2001). Endemism, species selection and the origin and distribution of the vascular plant flora of New Zealand. Journal of Biogeography, 28, 199–216. 10.1046/j.1365-2699.2001.00525.x

[ece37358-bib-0061] Millener, P. R. (1989). The only flightless passerine; the Stephen's Island Wren (*Traversia lyalli*: Acanthisittidae). Notornis, 36, 280–284.

[ece37358-bib-0062] Miller, H. C. , & Lambert, D. M. (2006). A molecular phylogeny of New Zealand’s Petroica (Aves: Petroicidae) species based on mitochondrial DNA sequences. Molecular Phylogenetics and Evolution, 40(3), 844–855. 10.1016/j.ympev.2006.04.012 16750641

[ece37358-bib-0063] Mitchell, K. J. , Wood, J. R. , Llamas, B. , McLenachan, P. A. , Kardailsky, O. , Scofield, R. P. , Worthy, T. H. , & Cooper, A. (2016). Ancient mitochondrial genomes clarify the evolutionary history of New Zealand's enigmatic acanthisittid wrens. Molecular Phylogenetics & Evolution, 102, 295–304. 10.1016/j.ympev.2016.05.038 27261250

[ece37358-bib-0064] Moyle, R. G. , Chesser, R. T. , Prum, R. O. , Schikler, P. , & Cracraft, J. (2006). Phylogeny and evolutionary history of old world Suboscine Birds (Aves: Eurylaimides). American Museum Novitates, 3544, 1–22.

[ece37358-bib-0065] Nei, M. (1987). Molecular evolutionary genetics. Columbia University Press.

[ece37358-bib-0066] Nielson, S. V. , Bauer, A. M. , Jackman, T. M. , Hitchmough, R. A. , & Daugherty, C. H. (2011). New Zealand geckos (Diplodactylidae): Cryptic diversity in a post‐Gondwanan lineage with trans‐Tasman affinities. Molecular Phylogenetics and Evolution, 59, 1–22. 10.1016/j.ympev.2010.12.007 21184833

[ece37358-bib-0067] Patel, S. , Waugh, J. , Millar, C. D. , & Lambert, D. M. (2010). Conserved primers for DNA barcoding historical and modern samples from New Zealand and Antarctic birds. Molecular Ecology Resources, 10, 431–438. 10.1111/j.1755-0998.2009.02793.x 21565042

[ece37358-bib-0068] Peakall, R. , & Smouse, P. E. (2005). GENALEX 6: Genetic analysis in Excel. Population genetic software for teaching and research. Molecular Ecology Notes, 6(1), 288–295. 10.1093/bioinformatics/bts460 PMC3463245 22820204

[ece37358-bib-0069] Posada, D. (2008). jModelTest: Phylogenetic model averaging. Molecular Biology and Evolution, 25, 1253–1256. 10.1093/molbev/msn083 18397919

[ece37358-bib-0070] Preston, S. A. , Briskie, J. V. , Burke, T. , & Hatchwell, B. J. (2013). Genetic analysis reveals diverse kin‐directed routes to helping in the rifleman *Acanthisitta chloris* . Molecular Ecology, 22, 5027–5039. 24033543 10.1111/mec.12448

[ece37358-bib-0071] Preston, S. , Dawson, D. A. , Horsburgh, G. J. , & Hatchwell, B. J. (2013). Characterisation of microsatellite loci in the Rifleman (*Acanthisitta chloris,* Acanthisittidae, AVES) and their predicted genome locations. Conservation Genetics Resources, 5, 555–560. 10.1007/s12686-012-9851-y

[ece37358-bib-0072] Pritchard, J. K. , Stephens, M. , & Donnelly, P. (2000). Inference of population structure using multilocus genotype data. Genetics, 155(2), 945–959. 10835412 10.1093/genetics/155.2.945 PMC1461096

[ece37358-bib-0073] Robertson, C. J. M. , Hyvonen, P. , Fraser, M. J. , & Pickard, C. R. (2007). Atlas of bird distribution in New Zealand 1999–2004, Vol. 2. Ornithological Society of New Zealand Inc.

[ece37358-bib-0074] Rogers, A. R. , & Harpending, H. (1992). Population growth makes waves in the distribution of pairwise genetic differences. Molecular Biology and Evolution, 9(3), 552–569. 1316531 10.1093/oxfordjournals.molbev.a040727

[ece37358-bib-0075] Rogers, A. R. , & McGlone, M. S. (1989). A postglacial vegetation history of the southern‐central uplands of North Island, New Zealand. Journal of the Royal Society of New Zealand, 19(3), 229–248. 10.1080/03036758.1989.10427179

[ece37358-bib-0076] Seutin, G. , White, B. N. , & Boag, P. T. (1991). Preservation of avian blood and tissue samples for DNA analyses. Canadian Journal of Zoology, 69, 82–90. 10.1139/z91-013

[ece37358-bib-0077] Tracy, L. N. , & Jamieson, I. G. (2011). Historical DNA reveals contemporary population structure results from anthropogenic effects, not pre‐fragmentation patterns. Conservation Genetics, 12, 517–526.

[ece37358-bib-0078] Trewick, S. A. , & Bland, K. J. (2011). Fire and slice: Palaeogeography for biogeography at New Zealand's North Island/South Island juncture. Journal of the Royal Society of New Zealand, 42(3), 153–183.

[ece37358-bib-0079] Trewick, S. A. , Pilkington, S. , Shepherd, L. D. , Gibb, G. C. , & Morgan‐Richards, M. (2017). Closing the gap: Avian lineage splits at a young, narrow seaway imply a protracted history of mixed population response. Molecular Ecology, 26, 5752–5772. 10.1111/mec.14323 28805283

[ece37358-bib-0080] Turbott, E. G. (1961). Birds. In W. M. Hamilton (Ed.), *Little Barrier Island (*Hauturu), Vol. 137. New Zealand DSIR Bulletin.

[ece37358-bib-0081] Van Oosterhout, C. , Hutchinson, W. F. , Wills, D. P. M. , & Shipley, P. (2004). MICRO‐CHECKER: Software for identifying and correcting genotyping errors in microsatellite data. Molecular Ecology Notes, 4(3), 535–538. 10.1111/j.1471-8286.2004.00684.x

[ece37358-bib-0082] Veitch, C. R. , Clout, M. N. , & Towns, D. R. (2011). Island invasives: Eradication and management. IUCN.

[ece37358-bib-0083] Wardle, P. (1963). Evolution and distribution of the New Zealand flora, as affected by quaternary climates. New Zealand Journal of Botany, 1(1), 3–17. 10.1080/0028825X.1963.10429318

[ece37358-bib-0084] Werle, E. , Schneider, C. , Renner, M. , Voker, M. , & Fiehn, W. (1994). Convenient single‐step, one tube purification of PCR products for direct sequencing. Nucleic Acids Research, 22(20), 4354–4355. 10.1093/nar/22.20.4354 7937169 PMC331970

[ece37358-bib-0085] Weston, K. , & Robertson, B. C. (2015). Population structure within an island archipelago: Strong signature of past climate change in the New Zealand rock wren (*Xenicus gilviventris*). Molecular Ecology, 24(18), 4778–4794. 26342535 10.1111/mec.13349

