# Supplementary material for: Genetic divergence between isolated populations of the North Island New Zealand Rifleman (Acanthisitta chloris granti) implicates ancient biogeographic impacts rather than recent habitat fragmentation
Source: Ecol Evol. 2021 May 4;11(11):5998–6014. doi: 10.1002/ece3.7358 (PMC8207446; doi:10.1002/ece3.7358)
Supplement: Supplementary file 1 — Appendix S1 [file ECE3-11-5998-s001.docx]

**Appendix**

**Supplementary Tables**

**Table A1**. Acanthisittid wren reference sequences used as outgroups for phylogenetic and coalescent analyses. * Extinct

| **Accession** | **Species** | **Scientific Name** | **Locus** | **Reference** |
| --- | --- | --- | --- | --- |
| AY325307 | Rifleman | *Acanthisitta chloris* (AC) | COI and  D-loop | ([Harrison et al., 2004](#_ENREF_2)) |
| KX369033 | Rock Wren | *Xenicus gilviventris* (XG) | COI | ([Mitchell et al., 2016](#_ENREF_3)) |
| KT229565 | Rock Wren | *Xenicus gilviventris* (XG) | D-loop | ([Weston, Taylor, & Robertson, 2016](#_ENREF_6)) |
| KT229566 | Rock Wren | *Xenicus gilviventris* (XG) | D-loop | (Weston et al., 2016) |
| KX369036 | Stout-Legged Wren | *Pachyplichas yaldwyni* (PY)* | COI | (Mitchell et al., 2016) |
| KX369034 | Lyall’s Wren | *Traversia lyalli* (TL)* | COI | (Mitchell et al., 2016) |
| KX369035 | Bush Wren | *Xenicus longipes* (XL)* | COI | (Mitchell et al., 2016) |

**Table A2.** Acanthisittid wren divergence dates (mya) used to calibrate Beast analysis (Mitchell et al. 2016). TL = *Traversia lyalli*, AC = *Acanthisitta chloris*, XG = *Xenicus gilviventris*, XL = *Xenicus longipe*s, PY = *Pachyplichas yaldwyni* (SD = Standard Deviation).

| **Divergence** | **Maximum Calibration Times** | | **Minimum Calibration Times** | |
| --- | --- | --- | --- | --- |
|  | **Date** | **SD** | **Date** | **SD** |
| TL \| AC, XG, PY, and XL | 32.9 | 3.2 | 16.6 | 2.5 |
| AC \| XG, TL, and XL | 17.3 | 0.7 | 7.0 | 1.0 |
| XL \| XG and PY | 14.2 | 1.5 | 5.7 | 1.0 |
| XG \| PY | 3.5 | 0.5 | 1.6 | 0.35 |

**Table A3:** Details of twelve microsatellite loci used in this study, derived from Preston et al. 2013a

| **Locus** | **Forward** | **Forward Primer Sequence** | **Reverse** | **Reverse Primer Sequence** | **Repeat** |
| --- | --- | --- | --- | --- | --- |
| Ach003 | HE598945 | F: TGAGCATGGTAACAACTACACAAG | Ach17B06 | R: AGGTGCTATGAGCATGAAACTG | AG |
| Ach008 | HE598950 | F: TTAGGTGGAGCCTATCCTTCG | Ach18F06 | R: GCTCCTTGTTCCTTACAGACATTC | ATCT |
| Ach010 | HE598952 | F: GAGGCAGTGTAACAGCAGACC | Ach21G08 | R: AGATGAGCTGAGGCTTGGAG | GT |
| Ach011 | HE598953 | F: TGCTGCTGGAATGGTTAAAG | Ach21H03 | R: GAGCACAGACATGACCAAATG | GT |
| Ach012 | HE598954 | F: CCAGCAATCTGCCTACCTG | Ach21H06 | R: CCACCTTCCTGAATCATAGTCC | GT |
| Ach014 | HE598956 | F: CAGGATGCTGCTATTCTTGATTC | Ach22B05 | R: ATCCCTGCGAGCAAATACC | ATCT |
| Ach018 | HE598960 | F: AGCTCAGGACAAGGTTCAGC | Ach22F12 | R: CTTCATCCCTGCTCATTGTG | GT |
| Ach019 | HE598961 | F: ACACGTCTGCTGCACTGTTC | Ach23A01 | R: GGGAAATTAGGGCAAACTCAG | AC |
| Ach024 | HE598966 | F: AGCATGCCCAGTACTTAACG | Ach23G05 | R: CCTGATAGTTTACACCAGGAATG | GATA |
| Ach026 | HE598968 | F: TCCGATTTGAACCTCTCTTATTTC | Ach23H08 | R: CTGACAGTCTCGGTCCCTTC | GT |
| Ach027 | HE598969 | F: TGACCTCACGTGCTCTCTGGG | Ach24B10 | R: AGTGCTAGTAACAACAGTGGCAGC | GT |
| Ach028 | HE598970 | F: GAGGTCTCTTCCAACCCAGTTGATTC | Ach24C02 | R: GAGCAGCTCCATGGTTCACCTTAC | AC) |

**Table A4.** BioGeoBears model weights and parameters

| **:** |  | | |  |  |  |  |  |  | **AIC and AICc model weights *within* each geographical model** | | | | |  |  |
| --- | --- | --- | --- | --- | --- | --- | --- | --- | --- | --- | --- | --- | --- | --- | --- | --- |
|  |  |  |  |  |  |  |  |  |  |  |  |  |  |  |  |  |
| **Geographic model** | **Base model** | **LnL** | **numparams** | **d** | **e** | **j** | **num_data** | **AIC** | **AICc** | **deltaAIC** | **deltaAICc** | **Relative likelihood (AIC)** | **Relative likelihood (AICc)** | **AIC model weight** | **AICc model weight** |  |
| H0 | DEC | -45.54 | 2 | 0.26 | 0.55 | 0 | 14 | 95.08 | 96.17 | 13.06 | 11.75 | 0.0015 | 0.0028 | 0.07% | 0.14% |  |
|  | DEC+J | -38.01 | 3 | 0.08 | 0.00 | 0.44 | 14 | 82.02 | 84.42 | 0.00 | 0.00 | 1.0000 | 1.0000 | 50.69% | 50.65% |  |
|  | DIVALIKE | -47.18 | 2 | 0.20 | 0.06 | 0 | 14 | 98.35 | 99.44 | 16.33 | 15.02 | 0.0003 | 0.0005 | 0.01% | 0.03% |  |
|  | DIVALIKE+J | -38.17 | 3 | 0.09 | 0.00 | 0.13 | 14 | 82.34 | 84.74 | 0.32 | 0.32 | 0.8521 | 0.8521 | 43.20% | 43.16% |  |
|  | BAYAREALIKE | -49.74 | 2 | 0.26 | 0.47 | 0 | 14 | 103.47 | 104.56 | 21.45 | 20.14 | 0.0000 | 0.0000 | 0.00% | 0.00% |  |
|  | BAYAREALIKE+J | -40.14 | 3 | 0.08 | 0.08 | 1.00 | 14 | 86.29 | 88.69 | 4.26 | 4.26 | 0.1187 | 0.1187 | 6.02% | 6.01% |  |
|  |  |  |  |  |  |  |  |  |  |  |  |  | **Total:** | 100.00% | 100.00% |  |
|  |  |  |  |  |  |  |  |  |  |  |  |  |  |  |  |  |
| H1 | DEC | -48.08 | 2 | 0.82 | 0.57 | 0 | 14 | 100.16 | 101.25 | 16.23 | 14.92 | 0.0003 | 0.0006 | 0.02% | 0.04% |  |
|  | DEC+J | -38.97 | 3 | 0.15 | 0.00 | 1.48 | 14 | 83.94 | 86.34 | 0.00 | 0.00 | 1.0000 | 1.0000 | 71.00% | 70.98% |  |
|  | DIVALIKE | -49.03 | 2 | 0.68 | 0.50 | 0 | 14 | 102.07 | 103.16 | 18.13 | 16.82 | 0.0001 | 0.0002 | 0.01% | 0.02% |  |
|  | DIVALIKE+J | -40.21 | 3 | 0.17 | 0.00 | 1.34 | 14 | 86.41 | 88.81 | 2.48 | 2.48 | 0.2900 | 0.2900 | 20.59% | 20.58% |  |
|  | BAYAREALIKE | -65.65 | 2 | 0.47 | 0.14 | 0 | 14 | 135.31 | 136.40 | 51.37 | 50.06 | 0.0000 | 0.0000 | 0.00% | 0.00% |  |
|  | BAYAREALIKE+J | -41.10 | 3 | 0.20 | 0.14 | 1.00 | 14 | 88.21 | 90.61 | 4.27 | 4.27 | 0.1181 | 0.1181 | 8.38% | 8.38% |  |
|  |  |  |  |  |  |  |  |  |  |  |  |  | **Total:** | 100.00% | 100.00% |  |
|  |  |  |  |  |  |  |  |  |  |  |  |  |  |  |  |  |
| H2 | DEC | -42.75 | 2 | 0.61 | 1.16 | 0 | 14 | 89.50 | 90.59 | 11.87 | 10.56 | 0.0026 | 0.0051 | 0.15% | 0.28% |  |
|  | DEC+J | -37.21 | 3 | 0.14 | 0.00 | 1.25 | 14 | 80.42 | 82.82 | 2.79 | 2.79 | 0.2482 | 0.2482 | 13.80% | 13.69% |  |
|  | DIVALIKE | -47.10 | 2 | 0.15 | 0.18 | 0 | 14 | 98.20 | 99.30 | 20.57 | 19.26 | 0.0000 | 0.0001 | 0.00% | 0.00% |  |
|  | DIVALIKE+J | -35.82 | 3 | 0.18 | 0.07 | 0.62 | 14 | 77.63 | 80.03 | 0.00 | 0.00 | 1.0000 | 1.0000 | 55.61% | 55.15% |  |
|  | BAYAREALIKE | -41.11 | 2 | 0.62 | 1.29 | 0 | 14 | 86.21 | 87.31 | 8.58 | 7.27 | 0.0137 | 0.0263 | 0.76% | 1.45% |  |
|  | BAYAREALIKE+J | -36.44 | 3 | 0.23 | 0.25 | 1.00 | 14 | 78.89 | 81.29 | 1.26 | 1.26 | 0.5336 | 0.5336 | 29.67% | 29.43% |  |
|  |  |  |  |  |  |  |  |  |  |  |  |  | **Total:** | 100.00% | 100.00% |  |
|  |  |  |  |  |  |  |  |  |  |  |  |  |  |  |  |  |
|  |  |  |  |  |  |  |  |  |  | **AIC and AICc model weights over *all* 18 models** | | | |  |  |  |
| **Geographic model** | **Base model** |  |  |  |  |  |  |  |  |  |  |  |  |  |  |  |
| H0 | DEC |  |  |  |  |  |  |  |  | 17.45 | 16.14 | 0.0002 | 0.0003 | 0.01% | 0.01% |  |
|  | DEC+J |  |  |  |  |  |  |  |  | 4.39 | 4.39 | 0.1113 | 0.1113 | 5.36% | 5.32% |  |
|  | DIVALIKE |  |  |  |  |  |  |  |  | 20.72 | 19.41 | 0.0000 | 0.0001 | 0.00% | 0.00% |  |
|  | DIVALIKE+J |  |  |  |  |  |  |  |  | 4.71 | 4.71 | 0.0948 | 0.0948 | 4.56% | 4.53% |  |
|  | BAYAREALIKE |  |  |  |  |  |  |  |  | 25.84 | 24.53 | 0.0000 | 0.0000 | 0.00% | 0.00% |  |
|  | BAYAREALIKE+J |  |  |  |  |  |  |  |  | 8.65 | 8.65 | 0.0132 | 0.0132 | 0.64% | 0.63% | 10.50% |
|  |  |  |  |  |  |  |  |  |  |  |  |  |  |  |  |  |
| H1 | DEC |  |  |  |  |  |  |  |  | 22.53 | 21.22 | 0.0000 | 0.0000 | 0.00% | 0.00% |  |
|  | DEC+J |  |  |  |  |  |  |  |  | 6.30 | 6.30 | 0.0428 | 0.0428 | 2.06% | 2.04% |  |
|  | DIVALIKE |  |  |  |  |  |  |  |  | 24.43 | 23.13 | 0.0000 | 0.0000 | 0.00% | 0.00% |  |
|  | DIVALIKE+J |  |  |  |  |  |  |  |  | 8.78 | 8.78 | 0.0124 | 0.0124 | 0.60% | 0.59% |  |
|  | BAYAREALIKE |  |  |  |  |  |  |  |  | 57.68 | 56.37 | 0.0000 | 0.0000 | 0.00% | 0.00% |  |
|  | BAYAREALIKE+J |  |  |  |  |  |  |  |  | 10.58 | 10.58 | 0.0051 | 0.0051 | 0.24% | 0.24% | 2.88% |
|  |  |  |  |  |  |  |  |  |  |  |  |  |  |  |  |  |
| H2 | DEC |  |  |  |  |  |  |  |  | 11.87 | 10.56 | 0.0026 | 0.0051 | 0.13% | 0.24% |  |
|  | DEC+J |  |  |  |  |  |  |  |  | 2.79 | 2.79 | 0.2482 | 0.2482 | 11.94% | 11.86% |  |
|  | DIVALIKE |  |  |  |  |  |  |  |  | 20.57 | 19.26 | 0.0000 | 0.0001 | 0.00% | 0.00% |  |
|  | DIVALIKE+J |  |  |  |  |  |  |  |  | 0.00 | 0.00 | 1.0000 | 1.0000 | 48.13% | 47.77% |  |
|  | BAYAREALIKE |  |  |  |  |  |  |  |  | 8.58 | 7.27 | 0.0137 | 0.0263 | 0.66% | 1.26% |  |
|  | BAYAREALIKE+J |  |  |  |  |  |  |  |  | 1.26 | 1.26 | 0.5336 | 0.5336 | 25.68% | 25.49% | 86.62% |
|  |  |  |  |  |  |  |  |  |  |  |  |  |  |  |  |  |
|  |  |  |  |  |  |  |  |  |  |  |  |  | **Total:** | 100.00% | 100.00% |  |

**Table A5**. Time-stratified dispersal matrices used in BioGeoBears analyses. I=Insular, W=Western, C=Central, ER=Eastern Ranges, EC=Eastern Coastal, S=Southern.

Dispersal Pattern C

|  | I | W | C | ER | EC | S |
| --- | --- | --- | --- | --- | --- | --- |
| I | 1 | 0.01 | 0.01 | 0.01 | 0.01 | 0.01 |
| W | 0.01 | 1 | 1 | 1 | 1 | 1 |
| C | 0.01 | 1 | 1 | 1 | 1 | 1 |
| ER | 0.01 | 1 | 1 | 1 | 1 | 1 |
| EC | 0.01 | 1 | 1 | 1 | 1 | 0.01 |
| S | 0.01 | 1 | 1 | 1 | 0.01 | 1 |

Dispersal Pattern B

|  | I | W | C | ER | EC | S |
| --- | --- | --- | --- | --- | --- | --- |
| I | 1 | 0.01 | 0.01 | 0.01 | 0.01 | 0.01 |
| W | 0.01 | 1 | 1 | 1 | 0.01 | 0.01 |
| C | 0.01 | 1 | 1 | 1 | 0.01 | 0.01 |
| ER | 0.01 | 1 | 1 | 1 | 0.01 | 0.01 |
| EC | 0.01 | 0.01 | 0.01 | 0.01 | 1 | 1 |
| S | 0.01 | 0.01 | 0.01 | 0.01 | 1 | 1 |

Dispersal Pattern A

|  | I | W | C | ER | EC | S |
| --- | --- | --- | --- | --- | --- | --- |
| I | 1 | 1 | 1 | 1 | 1 | 1 |
| W | 1 | 1 | 1 | 1 | 1 | 1 |
| C | 1 | 1 | 1 | 1 | 1 | 1 |
| ER | 1 | 1 | 1 | 1 | 1 | 1 |
| EC | 1 | 1 | 1 | 1 | 1 | 1 |
| S | 1 | 1 | 1 | 1 | 1 | 1 |
